# Water Drop Evaporation on Slippery Liquid-Infused Porous Surfaces (SLIPS): Effect of Lubricant Thickness, Viscosity, Ridge Height, and Pattern Geometry

**DOI:** 10.1021/acs.langmuir.3c00471

**Published:** 2023-04-27

**Authors:** Rana Üçüncüoğlu, H. Yildirim Erbil

**Affiliations:** Department of Chemical Engineering, Gebze Technical University, Gebze, 41400 Kocaeli, Türkiye

## Abstract

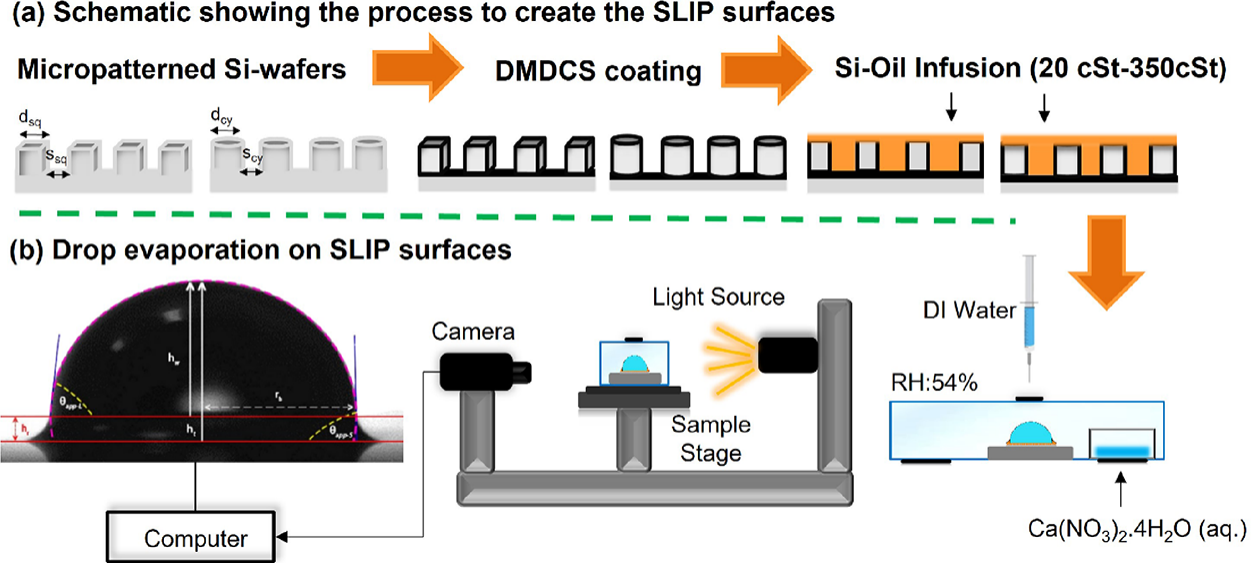

Sessile drop evaporation and condensation on slippery liquid-infused porous surfaces (SLIPS) is crucial for many applications. However, its modeling is complex since the infused lubricant forms a wetting ridge around the drop close to the contact line, which partially blocks the free surface area and decreases the drop evaporation rate. Although a good model was available after 2015, the effects of initial lubricant heights (*h*_oil_)_i_ above the pattern, and the corresponding initial ridge heights (*h*_r_)_i_, lubricant viscosity, and solid pattern type were not well studied. This work fills this gap where water drop evaporations from SLIPS, which are obtained by infusing silicone oils (20 and 350 cSt) onto hydrophobized Si wafer micropatterns having both cylindrical and square prism pillars, are investigated under constant relative humidity and temperature conditions. With the increase of (*h*_oil_)_i_, the corresponding (*h*_r_)_i_ increased almost linearly on lower parts of the drops for all SLIPS samples, resulting in slower drop evaporation rates. A novel diffusion-limited evaporation equation from SLIPS is derived depending on the available free liquid–air interfacial area, *A*_LV_, which represents the unblocked part of the total drop surface. The calculation of the diffusion constant, *D*, of water vapor in air from (d*A*_LV_/d*t*) values obtained by drop evaporation was successful up to a threshold value of (*h*_oil_)_i_ = 8 μm within ±7%, and large deviations (13–27%) were obtained when (*h*_oil_)_i_ > 8 μm, possibly due to the formation of thin silicone oil cloaking layers on drop surfaces, which partially blocked evaporation. The increase of infused silicone oil viscosity caused only a slight increase (12–17%) in drop lifetimes. The effects of the geometry and size of the pillars on the drop evaporation rates were minimal. These findings may help optimize the lubricant oil layer thickness and viscosity used for SLIPS to achieve low operational costs in the future.

## Introduction

Inspired by the Nepenthes pitcher plant, slippery liquid-infused porous surfaces (SLIPS) were developed by Aizenberg and co-workers in 2011.^1^ SLIPS are also termed as “lubricant infused surfaces” (LIS) by other authors. SLIP surfaces can be obtained by infusing a water immiscible lubricant having low surface free energy onto a textured solid to give a liquid lubricant over layer having a smooth and continuous surface, resulting in a pinning-free and mobile three-phase contact line of water and some other liquid drops placed on it. SLIP surfaces have a very low contact angle hysteresis for the drops. In practice, the streaming lubricant layer can spontaneously flow into the textured substrate in a very short period of time, and SLIP surfaces have better self-healing properties than superhydrophobic surfaces since the lubricant can automatically fill the voids or previously dried locations on the surface. Many good review articles were published on the synthesis and properties of SLIPS in the last decade.^2−8^ SLIP surfaces have the potential to be applied in various industrial fields such as condensation heat transfer,^7,9−14^ anti-icing,^15−20^ medical,^21−24^ marine biofouling,^25−27^ and hydrodynamic drag reduction^28−31^ and others. Thermodynamical description of infusing a micro- or nanostructured solid surface with a low–surface tension liquid was given in terms of balances of interfacial energies (lubricant–solid γ_LS_, lubricant–vapor γ_LV_, and solid–vapor γ_SV_) and the geometrical parameters characterizing the surface textures.^32−34^ A stable lubricant film can form in the solid texture if it is energetically favorable to spread into the texture in comparison with forming a discrete drop on the same structure.

Drop evaporation is an important topic both in academia and industry.^35−38^ Without the presence of a convective air current, a drop evaporates with a simultaneous heat and mass transfer operation via a diffusion limited evaporation process where heat is transferred by conduction from warm air to the drop surface and phase change happens with the transformation of liquid into vapor and transfer of the vapor into the air by diffusion. The air which is in the immediate vicinity of the drop becomes saturated with the vapor molecules that are liberated from the liquid, and thus the rate limiting step is the diffusion of vapor molecules away from the drop to a far field after the saturation of vapor pressure. Both the theoretical and experimental studies indicate that several different factors affect the evaporation rates of sessile drops: (i) initial contact angle and drop shape;^39−49^ (ii) properties of the solid substrate including substrate roughness,^50,51^ heat conductivity;^52,53^ (iii) nature of the liquid, including its surface tension and volatility;^54^ (iv) properties of the surrounding gas, including atmospheric pressure,^55,56^ humidity,^57−59^ and the temperature of each phase.^60,61^

The evaporation of small water sessile droplets (∼2 mm in diameter) with an apparent contact angle greater than 90° on SLIP surfaces was first investigated using patterned substrates made of square pillars of hydrophobized SU-8 photoresist and silicone oil as lubricant in 2015.^62^ In this pioneering work, it was reported that the droplet profiles on SLIPS were more complicated than that of on the solid surfaces where the spherical cap drop profile was modified by a wetting ridge of lubricant close to the contact line around the droplet due to the equilibrium of the interfacial forces at the line of contact between the droplet, the lubricant, and air (as expected by a Neumann triangle). It was found that the drop evaporation process followed an ideal constant contact angle (CCA) mode where the apparent contact angle was defined from the intersection of the substrate profile with the droplet spherical cap profile. Using *r*_o_^2^–time plots, where *r*_o_ was the apparent contact radius of the drop, which was measured at the point of inflection of the drop profile, a diffusion limited drop evaporation model was developed to account for the screening effect of the wetting ridge against evaporation.^62^ The formed wetting skirt around the droplet was found to limit the droplet liquid–vapor surface area available for the evaporation, and the apparent contact surface area was linearly dependent on time. The diffusion coefficients for water vapor in air were calculated using the developed drop evaporation model, and very good correlation within an average of 4% of reference values was determined. Moreover, a stick–slip regime was detected when the water droplet was in contact with the square pillar tops with the decrease of the lubricant level; however, such cases were excluded from the drop evaporation analysis since the droplets did not demonstrate a CCA mode for these situations.^62^

The comparison of the total drop evaporation times between flat, superhydrophobic, and SLIP surfaces was reported in another study.^63^ The total evaporation times of 5 μL water droplets on SLIPS, which were obtained by infusing fluorinated lubricant (Krytox 140) onto a hydrophobized nanostructured Cu surface, were determined and compared with the total evaporation times of the water droplets of the same volume on flat hydrophobic Cu surfaces and superhydrophobic hydrophobized nanostructured Cu surfaces. The longest evaporation time was found to be on the SLIP surfaces, and the reduction in evaporation rate was attributed to the evaporative cooling at the droplet interface due to the slow reduction in the height of an evaporating water droplet with time. When the water droplet height was large, a longer thermal resistance path was present, which extended the total evaporation time. In addition, SLIP surfaces had lower thermal effusivity (square root of the product of the material’s thermal conductivity, density, and specific heat capacity) than others, and their capacity to exchange the thermal energy with their surroundings was low.^63^

McHale and co-workers extended their initial drop evaporation study on SLIPS^62^ with two new publications.^64,65^ In the first of these articles, they prepared smooth (non-textured) “slippery omniphobic covalently attached liquid-like” (SOCAL) surfaces, where covalently attached polydimethylsiloxane chains were grafted onto a flat glass substrate in order to remove contact-line pinning and stick–slip behavior during the evaporation of sessile water droplets.^64^ No wetting ridge was present around water droplets on the SOCAL surfaces, resulting in high contact line mobility, and the initial contact-angle hysteresis was found to be very small (between 0.4 and 1.1°). The CCA mode of evaporation was observed on SOCAL surfaces for most of the evaporation time at constant relative humidity (RH) across the range of 10–70% at 25 °C. When the CCA mode of the diffusion-limited evaporation model was applied, the calculation of the diffusion coefficient of water vapor in air from the experimental data was accurate to within 2% of the value reported in the literature.^64^ In the second article, water drop evaporation experiments on both SOCAL and SLIP surfaces (using silicone oil as lubricant) were carried out with the simultaneous application of electrowetting.^65^ Initial water droplet contact angles between 67 and 105^o^ (both hydrophilic and hydrophobic) could be obtained with the use of the electrowetting-on-dielectric method on these surfaces, and it was assumed that the presence of electric charges would not considerably influence the evaporation rate of sessile droplets since they are stored at the solid–liquid interface. The CCA mode of drop evaporation was seen in most of the cases, and the contact angle dependence of water droplet lifetime was found to be relatively insensitive to the initial contact angle (within 10%) for the contact angle range of 40–180°. It was reported that the diffusion-controlled evaporation model of sessile droplets can be successfully used to estimate the water vapor diffusion coefficient in these conditions.^65^

The evaporation of very small droplets from SLIPS was reported in another study, where three processes, dropwise condensation of water vapor, droplet evaporation, and droplet self-propulsion were simultaneously investigated.^66^ SLIP surfaces were prepared by treating glass slides with a layer of commercial superhydrophobic agent (Glaco Mirror Coat) and using Krytox GPL as the infusing lubricant. For small droplet evaporations on SLIPS in the CCA mode, the ridge height-to-droplet size ratio was found to increase significantly when the droplet diameter was greater than 130 μm and the maximum oil ridge height remained approximately constant due to the limited oil supply from the lubricant. When the droplet size decreased, the maximum oil ridge height reduced for droplets with diameters less than 130 μm.^66^

In a recent study, the importance of the “wetting ridge” and “cloaking” of both pure water and binary solution (water–ethanol) droplets on flat SLIP surfaces was reported.^67^ SLIP surfaces were prepared by spin-coating silicone oil (350 cSt) on octadecyltrichlorosilane self-assembled monolayer coated flat glasses. Two different thicknesses of the silicone oil layer (25 and 5 μm) were prepared by spin-coating oil at 1000 and 5000 rpm, respectively. The initial volume of the all the drops was kept constant at 4 μL, and an environmental chamber at constant temperature of 22 °C and RH of 38 ± 2% was used. It was shown that wetting ridge dynamics play a major role and the wetting ridge height of an evaporating drop varies non-monotonically compared to the drop height, which also decreases monotonically during the evaporation of drops from the liquid–vapor interface. Wetting ridges around a drop completely prevented the evaporation from that region, and drops could evaporate only through the ridge-free area. After the deposition of the droplet on the lubricant surface, the wetting ridge height increased rapidly toward the equilibrium value, and later, as the drop continues to evaporate, the wetting ridge height increased slowly to its maximum value due to the moving contact line. Finally, the ridge height started to decrease along with the drop height, which continued till the end of the evaporation.^67^ Moreover, the increase of the lubricant thickness from 5 to 25 μm resulted in a considerable increase in both initial and maximum ridge heights. Thus, the thickness of the lubricating liquid on a solid is important since it affects many parameters such as the apparent contact angle, drop height, and wetting ridge height. For the same water drop volumes, the total evaporation time was longer when the drop was placed on a thick lubricating film compared with a thin lubricating film. However, the experimental and theoretical results based on the diffusion-limited evaporation model were not in very good agreement for these drop evaporation studies, which was attributed to the presence of a wetting ridge and the cloaking ultrathin lubricant film on the drop.^67^

In a recent article, the evaporation of water droplets with 10 μL volume from SLIP surfaces, which were obtained by the replication of rose petals (sticky hydrophobicity) was investigated using a cross-linked polydimethylsiloxane substrate.^68^ The hierarchically patterned rose-petal substrates had many secondary nanofolds on them that decorated the primary conical micropillars and imparted stickiness to the high-viscosity silicone oil (1000 cSt) layer and suppressed hemi-wicking even at low oil layer thicknesses. When the lubricant oil thickness was reduced, the maximum height of the wetting ridge decreased with the corresponding reduction in the fractional coverage of the droplet surface by the wetting ridge and caused the faster evaporation of water drops on SLIPS. A small effect of the silicone oil layer viscosity on an evaporating drop on SLIPS was reported, where the lubricant oil layer viscosity was only significant when the oil layer thickness was comparable to the height of the substrate features. It was speculated that the amount of oil available for drop cloaking would be likely to increase with the increase in the oil layer thickness.^68^

In this work, we investigated pure water drop evaporation from SLIP surfaces obtained by infusing silicone oil having two different viscosities (20 and 350 cSt) onto hydrophobized microscopic patterns prepared on Si wafers having both cylindrical and square pillars under controlled RH and temperature conditions. In 2015, drop evaporation studies with the CCA mode were carried out on SLIP surfaces, which were prepared by infusing silicone oil (20 cSt) onto hydrophobized square microscopic pillars, and very good correlation within an average of 4% of reference values was found for the diffusion coefficients of water vapor in air, *D*, by applying the diffusion-limited drop evaporation model.^62^ However, some conflicting results were reported in the literature where the experimental and theoretical results based on the diffusion-limited CCA evaporation model were not in very good agreement for drop evaporation on SLIP surfaces,^67^ and it is important to clarify this point. Previously, only patterns with square prism pillars were used in the drop evaporation on SLIPS studies,^62^ and thus, we tried to investigate the effect of the use of patterns with cylindrical pillars on the drop evaporation rates on SLIPS in comparison with the patterns with square prism pillars. For this purpose, we selected the Cassie–Baxter area and linear lubricant fractions of our cylindrical and square prism patterns to be identical with the micropatterned samples, which were given in ref (62). On the other hand, it is reported several times that lubricant thickness has a considerable effect on the wetting ridge height, apparent contact angle, and apparent contact radius and contributes considerably to the rate of drop evaporation, although only a few experimental data are available. Thus, we varied the lubricant film thickness above the pillar tops incrementally in our experiments and studied the effect of the lubricant film thickness on the ridge height and drop evaporation rates for SLIPS having both square and cylindrical pillars. We also derived an equivalent but different diffusion-limited CCA evaporation equation for this purpose depending on the available drop–air interfacial area free of lubricant skirts.

## Theory

Picknett and Bexon derived the expressions for the mass loss of a diffusion-controlled evaporation of a methyl acetoacetate drop on a polytetrafluoroethylene (PTFE) surface in still air in 1977.^39^ Two pure modes of drop evaporation were determined: (i) constant contact angle mode (CCA) with diminishing contact area and (ii) constant contact area or radius (CCR) mode with diminishing contact angle. A mixed type was also reported, where the mode would change from one to the other at later courses of evaporation.^39^ In general, the CCR mode is the dominating evaporation mode for water and many other drops on solids, especially when the initial contact angle is less than 90° where contact-line pinning has been observed to some degree.^36−38^

On the other hand, the occurrence of the CCA mode of evaporation on a flat substrate is a rare process since it requires complete mobility of the three-phase contact line around the drop with the same contact angle; however, most of the ordinary flat solid surfaces are rough to some extent and result in contact-line pinning. The drop profile preserves that of a spherical cap shape with a decreasing drop height (*h*) and contact area between the liquid and solid (*A*_SL_ = π*r_b_^2^*), while θ retains a constant value during the CCA evaporation mode. Thus, the square of the base radius of the droplet (*r*_b_^2^) decreases linearly in time.^36,47,62,69,70^ The CCA mode of drop evaporation for contact angles less than 90° was seen during the first stage of evaporation of *n*-butanol, toluene, *n*-nonane, and *n*-octane drops on PTFE by applying video microscopy and digital image analysis techniques.^47^ The decrease of *r*_b_^2^ and *h*^2^ was found to be linear with time, and the *f*(θ) factor, which was derived by Picknett and Bexon for diffusion-limited evaporation, was also included in the equations in that study. *f*(θ) represents the effect of the presence of the solid substrate beneath the drop, which hinders the drop evaporation rate. This effect can be understood by a simple comparison: if the evaporation rate of a fully spherical drop with a contact angle of 180°, which is located on a flat substrate, is compared with the evaporation rate of a hanging fully spherical drop of the same volume surrounded by free space, the latter would evaporate faster because of the lack of the solid plane barrier preventing the vapor, which can diffuse downward. The *f*(θ) factor was found to be a function of contact angle of the spherical cap profile, and an exact closed-form solution for the diffusion-limited evaporation of sessile drops was provided by Picknett and Bexon as polynomial fits to *f*(θ), covering the full contact-angle range.^39,47^ The equations for the drop evaporation rate, which was derived in ref (47), are given below:
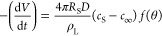
1where *t* is the time (s), *R*_S_ is the radius of the spherical drop, *D* is the diffusion coefficient (m^2^/sec), *c*_S_ is the concentration of vapor at the sphere surface (at *R*_S_ distance, kg/m^3^), *c*_∞_ is the concentration of the vapor at infinite distance (*R*_∞_ distance, kg/m^3^), ρ_L_ is the density of the drop substance (kg/m^3^), and *f(*θ*)* is a function of contact angle of the spherical cap shaped drop. From geometrical and trigonometrical considerations,
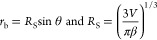
2where
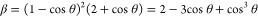
3

It was given that^47^
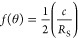
4where
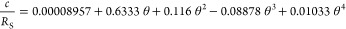
5for 0.175 ≤ θ ≤ π in radians (10° ≤ θ < 180°)^39^ and by combining eqs 1 and 2, one obtains
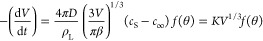
6where  and is a constant independent of time and drop volume. Equation 6 can be readily integrated from the *V*_i_ (initial drop volume) when *t* → 0 and *V* when *t* → *t*, giving
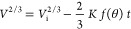
7

Since  from eq 2, by combining it with eqs 3 and 7, one obtains^70^
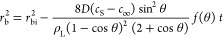
8

On the other hand, the interfacial area of a three-dimensional spherical liquid drop at the liquid/vapor interface (*A*_LV_) is given from trigonometry as
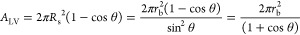
9

By combining eqs 8 and 9, one obtains
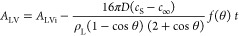
10

The diffusion constant, *D*, is assumed to be independent of the partial density of the vapor of the evaporating liquid for the low concentrations of the evaporating vapors by definition.^36,70−73^ The concentration of vapor (or vapor density) *c* can be given by using the ideal gas laws
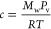
11where *M*_w_ is the molecular weight, *P*_v_ is the vapor pressure of the evaporating substance, *R* is the gas constant, and *T* is the absolute temperature in Kelvin. By combining eqs 10 and 11, one obtains
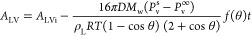
12where *P*_v_^s^ is the saturated water vapor pressure at the drop surface and *P*_v_^∞^ is the water vapor pressure in the ambient still air far from the drop surface for water drop evaporations. The relative humidity of the medium (RH) is related with *P*_v_^s^ as given below:
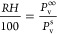
13

By combining eqs 12 and 13, one obtains
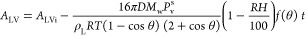
14

There are two options for eq 14. For an organic liquid drop, the vapor concentration in ambient air at ∞ distance would be zero; however, this is not valid for a water drop since the water vapor is already present in a definite amount in the ambient atmosphere in open-air conditions. The water vapor concentration and actual water vapor pressure of the medium can be calculated if RH and the ambient temperature are known using eqs11 and 13. It should be noted that the temperature dependencies of some parameters are different in eq14. For example, *D* is dependent on the ambient temperature, *P*_v_^s^ is dependent on the surface temperature of the drop, which is generally less than the ambient temperature due to cooling of the drop surface during evaporation by the transfer of latent heat (considerable surface cooling was reported in some articles depending on high evaporation rates)^36,42,44,70−74^ and the liquid density, ρ_L_, is dependent on the (average) bulk drop temperature, which is between the ambient and the drop surface temperatures since there is a temperature gradient inside the drop.^36^ Considering all of these factors, it is possible to estimate the experimental value of the diffusion coefficient of water vapor in air from eq 14 and evaluate the efficiency of the drop evaporation experiments by comparing the experimental *D* value with the published *D* values in the literature.
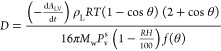
15

Unfortunately, this equation cannot be directly applied to the drops that are evaporated from SLIP surfaces, where the spherical cap drop profile was modified by a wetting ridge of lubricant due to the equilibrium of the interfacial forces at the line of contact between the droplet, the lubricant and air.

**Figure 1:**
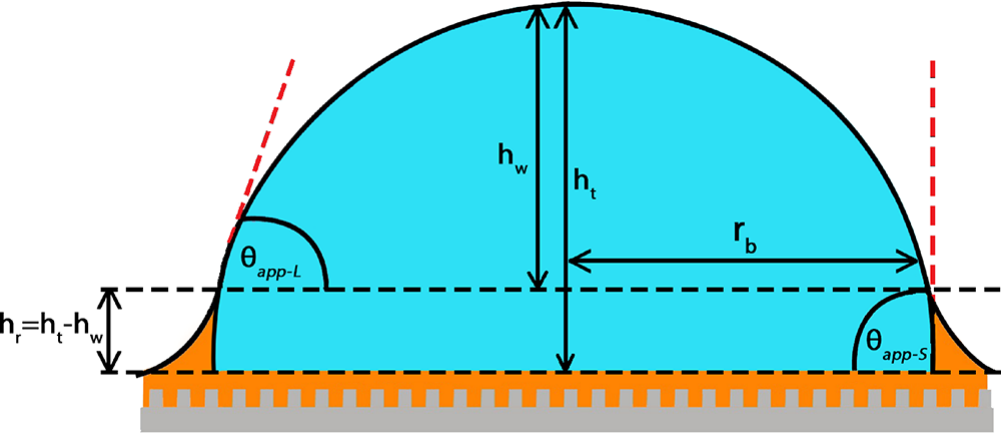
Schematic description of the cross section of the water drop on the silicone oil-infused lubricant layer over the micropatterned SLIP surface, showing defined parameters. The wetting ridge is formed around the drop by the balance of interfacial forces between the silicone oil and the evaporating water drop.

For SLIPS, a diffusion-limited drop evaporation model in the CCA mode was developed to account for the screening effect of the wetting ridge against evaporation.^62^ The sessile drop sat on the lubricant layer and it had no direct contact with the underlying solid surface, and thus, there were two different contact angles where the apparent contact angle at the lubricant ridge height (*h*_r_) level, θ_app-L_, was defined at the intersection between the infusing lubricant, water, and the surrounding air, while the other apparent contact angle, θ_app-S_, was defined at the horizontal lubricant level just above the solid, at the intersection between the infusing liquid, water, and the solid, as seen in Figure 1.^62^

The lubricating liquid at the *h*_r_ level, which was above the solid level, reduced the liquid–vapor interfacial area of the drop by covering the bottom of the drop with a nonvolatile lubricant layer. It was found that the apparent contact surface area of the solid–liquid (*A* = π*r*_b_^2^) interface was linearly dependent on time, and *r*_b_^2^–time plots were used to calculate the *D* constant, where *r*_b_ is the apparent contact radius of the water drop, which was measured at the point of inflection of the drop profile at the *h*_r_ level.^62^ By applying the same approach to eq 15, one obtains
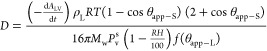
16

Although mathematically equivalent with the equations using *r*_b_^2^–time plots,^62,65−67^equation 16 is more advantageous than the previous one due to its intuitive nature and advantage for visual perception since the d*A*_LV_/d*t* parameter represents the free interfacial area for drop evaporation between liquid and air, which is not blocked by the lubricant ridge around the drop. We checked the validity of this equation by using the published data of ref (62) and obtained very similar *D* values. Then, eq 16 was employed for all the drop evaporation experiments on the SLIP surfaces in this study.

## Experimental Section

### Materials

Dimethyldichlorosilane (DMDCS) was obtained from FLUKA, hexane was obtained from MERCK, silicone oils having kinematic viscosities of 20 and 350 cSt were obtained from Sigma Aldrich, and Ca(NO_3_)_2_·4H_2_O and pure water (spectroscopic grade) were obtained from MERCK.

### Patterning Silicone Wafers by DRIE

Silicon wafers with regular square prism and cylindrical pillar structures with varying sizes and having an average height of 31 ± 2 μm were obtained the by the deep reactive ion etching (DRIE) method using single-side-polished silicon wafers.^75^ Initially, a photoresist material was coated on Si wafers by spin-coating and annealed at 115 °C for a minute with the following exposure to UV light using a photomask for 5 s. The samples were etched in a developer solution to remove the unnecessary photoresist layer on the Si wafer. Later, the uncoated Si surface was etched thoroughly using the DRIE technique down to around 31 μm depth. Si wafers were finally cleaned by O_2_ plasma after the etching process to remove the remaining photoresist material.^75^

### Hydrophobization of the Silicone Patterns

Patterned silicon wafers were cleaned by keeping them in chromic acid solution for 8 h, washed with distilled water several times, and dried in an oven. Then, these patterns were hydrophobized in the vapor phase by coating their surfaces with DMDCS, which was diluted 2:1 (v/v) in hexane solvent in a cup. Both the sample and DMDCS solution were kept in a desiccator at 65 °C in an oven, where the vapor phase reaction was carried out overnight. After the silane coating, the wafers were washed sequentially with toluene, ethanol, ethanol/distilled water solution 1:1 (v/v), and distilled water and dried in an oven at 120 °C for an hour.

### Formation of SLIPS by Infusing Silicone Oil on Patterned Silicon Wafers

DMDCS-coated silicon wafers were infused with two silicone oils having different kinematic viscosities (20 and 350 cSt) to obtain SLIPS. Initially, the empty masses of the silicon wafers were determined following the dropwise addition of a pre-determined amount of silicone oil (8–14 mg) onto the DMDCS-coated silicon wafer using a glass Pasteur pipette. Later, the infused silicone pattern samples were placed on glass slides and kept laterally for 24 h to ensure that the silicone oil was well spread on the SLIPS sample surface, giving an uniform oil thickness.

### Optical Microscopy

A Nikon ECLIPSE LV 100 Optical Microscope was used to obtain the plan images of the silicon wafers.

### Contact Angle Measurement

A KSV CAM 200 instrument was used for the contact angle measurements. A motorized dispenser controlled with a computer was used to form pure water drops of 5 μL on the SLIP surfaces for the measurement of apparent contact angles (θ_app-L_ and θ_app-S_) from the drop profiles. The drop dimensions and contact angles were determined by the software of the instrument after recording the drop profile. Advancing contact angle (θ_*a*_) and receding contact angle (θ*_r_*) measurements were also carried out on the DMDCS-coated flat and patterned substrates. For θ_a_ measurement, initially, 3 μL of a water drop was formed where the needle connected with the dispenser was kept in the drop. Then, 5 μL of extra water was added to the same drop with a flow rate of 0.1 μL/s through the needle, the images were recorded during this period, and the maximum value of the contact angle was determined as θ_a_. For the θ_r_ measurements, initially, 8 μL of a water drop was formed on the sample, and the drop volume was reduced to 3 μL with a suction rate of 0.1 μL/s through the needle. The contact angle change with time was plotted, and the minimum value of the contact angle was determined as θ_r_. The contact angle hysteresis (CAH) value is the difference between the θ_a_ and θ_r_.

### Calculation of Silicone Oil Heights over the Patterns

The volume of the empty space for oil infusion on the hydrophobized silicone patterns was calculated from the geometry of the pillars. The height of both the cylindrical and square pillars was assumed to be 31 μm on average, and all wafers were 2 cm × 2 cm in size. After calculating the number of pillars on each sample, the total volume occupied by the solid pillars on the sample was determined, and the void volume was found by subtracting this from the total volume of the rectangular prism of 2 cm × 2 cm area with an average thickness of 31 μm. The required masses of the minimum amount of silicone oils to fill the void volume of the samples were calculated from the densities of silicone oils of 20 and 350 cSt, which were 950 and 968 kg/m^3^, respectively. The tops of the pillars were exposed to air and put in contact with the basement of the water drop if the silicone oil volume was less than the minimum void volume. We avoid such a situation in all of our experiments, similar to the study given in ref (62), by monitoring the disappearance of the pillar tops by the incremental addition of silicone oil while checking the surface by optical microscopy. The height of the silicone oil layer (*h*_oil_) describes the height of silicone oil level above the pillar top level. The *h*_oil_ value was calculated gravimetrically using the added mass of silicone oil onto sample total area (400 mm^2^) by using their densities. For this purpose, silicone oil was added incrementally in definitive amounts on the patterned SLIPS samples in a Precisa 320-XB balance. The uncertainty in the calculated *h*_oil_ values was between ±0.67 and 1.25% since the sensitivity of the balance was 0.1 mg over a total mass of 15 to 8 mg added silicone oil. The SLIPS samples were then placed in the plexiglass chamber before the drop evaporation studies. Durability tests were carried out on the silicone oil height levels, and it was determined that no change occurred in the initial (*h*_oil_) level in 24 h for both 20 and 350 cSt Si-oils by evaporation or leaking from the S-1, S-2, and S-3 SLIPS samples.

### Experimental Determination of Ridge Height of Water Drop on SLIPS

**Figure 2:**
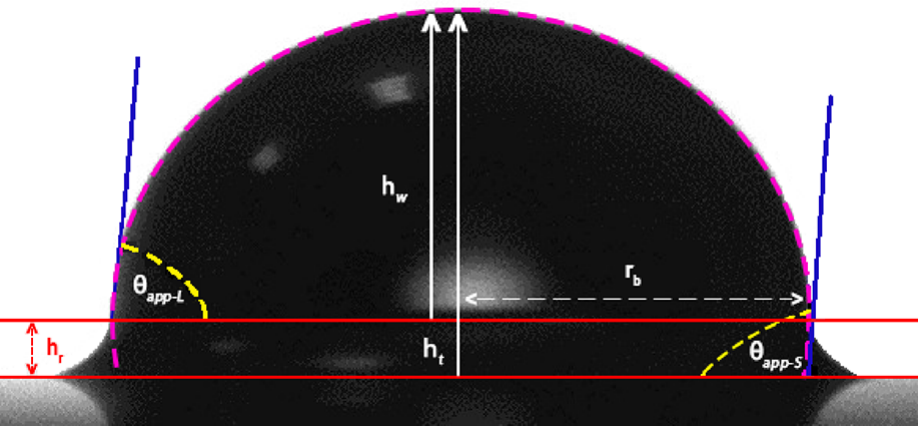
Side view profile of the spherical cap-shaped sessile water drop on the silicone oil-infused micropatterned SLIP surface showing the presence of the inflection point, which was used to determine the contact radius (*r*_b_), apparent contact angle at the liquid three-phase contact line (θ_app-L_), and ridge height (*h*_r_). The apparent contact angle at the solid three-phase contact line (θ_app-S_) was calculated by applying a numerical fitting procedure to obtain the intersection of the circular arc of the main drop profile with the baseline of the drop.

After locating a water drop of 5 μL in volume on the freshly prepared SLIP surface and removing the needle, the apparent contact angle (θ_app-L_) was determined at the liquid three-phase contact line (L-TPCL) at the inflection point of the lubricant–water–air phases as seen in Figure 1. The level of the silicone oil layer could be clearly seen near the drop base and was taken as a reference point for the total drop height (*h*_t_) as well as for the water drop height above the oil ridge (*h*_w_). The difference between these two drop heights (*h*_t_ – *h*_w_) is determined as the ridge height (*h*_r_) as seen in the indicative drop profile image given in Figure 2.

Later, the apparent contact angle (θ_app-S_) was determined at the solid three-phase contact line (S-TPCL) at the intersection point of the oil–water–solid phases. For this purpose, the intersection of the circular arc of the main drop profile with the baseline of the drop was constructed by applying a fitting procedure using the software of an KSV CAM 200 Contact Angle instrument, as seen in Figure 2. It is known that the continuation of the spherical drop contour from the exposed surface through the surface, hidden by the ridge, can be applied when the lubricant oil cloaks the drop especially for the case where the water drop sits on the solid patterns due to the energy balance of the three fluid phases in contact with the solid substrate.^76−78^

### Drop Evaporation Experiments

All the water drop evaporation experiments were carried out in a transparent plexiglass closed cell at a constant RH of 54 ± 1% using a saturated calcium nitrate tetrahydrate [Ca(NO_3_)_2_·4H_2_O] hygrostat salt solution at 25 ± 1 °C temperature. The temperature and RH of the cell were monitored by a TFA-Dostmann digital thermohygrometer (cat. no.: 30.5005, Germany) placed inside the cell. The SLIPS samples that were infused by silicone oils were placed in the transparent plexiglass test cell and kept there for 24 h to reach the equilibrium RH and temperature in the cell, and then 5 μL of spectroscopic grade pure water was manually injected with a rotating pump syringe (Hamilton) onto the substrate surface through a medical rubber stopper placed on the plexiglass cell. An Attention-THETA contact angle instrument was used to monitor the drop profiles during the evaporation experiments. Image recording was performed at 30 s intervals throughout the entire drop evaporation time between 2900 and 5200 s. The change of the drop contact radius (*r*_b_) and apparent contact angles (both θ_app-S_ and θ_app-L_) with time was monitored. Drop evaporation experiments were carried out three times on each pattern to test the repeatability of the data. Four sets of experiments were implemented with the incremental increase of the silicone oil layer thickness to determine the effect of silicone oil heights on the drop evaporation rates and lifetimes of the water drops. The recorded drop profile images were analyzed using the software of the instrument.

## Results and Discussion

### Structure and Properties of the Microscopic Patterns

**Figure 3:**
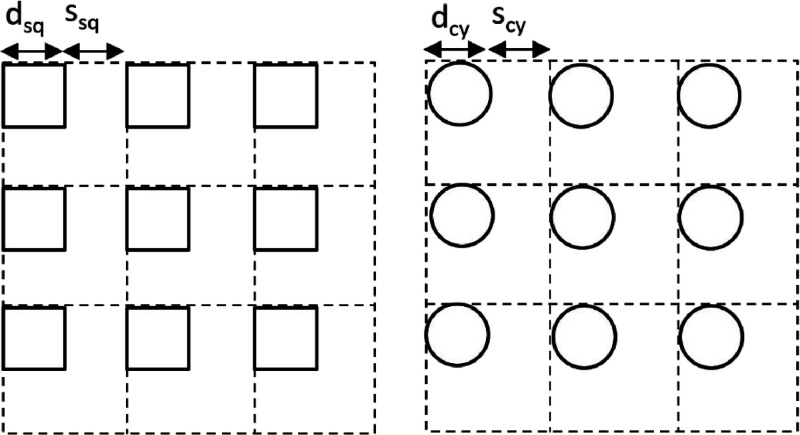
Schematic description of the square and cylindrical microscopic patterns from plan view.

Two square microscopic patterns and one cylindrical microscopic pattern were used in the experiments. Their schematic description from plan view is given in Figure 3. The separation distance, *s*, is the distance between two pillars from one edge to the other, *d*_sq_ shows the side length of the square pillar, and *d*_cy_ is the diameter of the cylindrical pillar.

The Cassie–Baxter solid fraction parameter for square prism pillars was calculated from geometric considerations using  and for cylindrical pillars using  equations.^79^ Similarly, the Wenzel roughness parameter for square prism pillars was calculated using [*r*_sq_^w^ = (*d*_sq_ + *s*_sq_)^2^ + 4*d*_sq_*h*_sq_/(*d*_sq_ + *s*_sq_)^2^] and that for cylindrical pillars was calculated using [*r*_cy_^w^ = (*d*_cy_ + *s*_cy_)^2^ + π*d*_cy_*h*_cy_/(*d*_cy_ + *s*_cy_)^2^] equations.^80^ The linear lubricant fraction *l*_f_ of the micropatterns is given as [*l*_f_ = *s*/(*s + d*)], which shows the ratio of the texture gap width to the unit length.^62^

**Figure 4:**
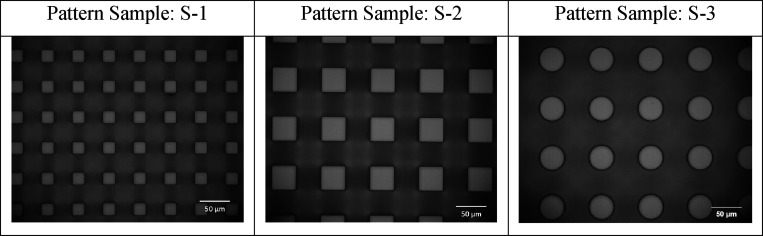
Optical microscopy images (500×) of the used patterns.

The optical microscopy images of the used patterns are given in Figure 4, and their geometrical and wetting properties, including the apparent (θ_app_), advancing (θ_a_), and receding (θ_r_), water contact angle results, are given in Table 1.

**Table 1:** Geometrical and Water Wetting Properties of the Patterned Hydrophobic Samples

sample	pillar type	*d* (μm)	*s* (μm)	*l*_f_	*f*_s_^CB^	*r*_w_	θ_app_ (^o^)	θ_a_ (^o^)	θ_r_ (^o^)	CAH (^o^)
S-1	square prism	20	30	0.6	0.160	2.024	155 ± 2	164 ± 1	150 ± 2	14 ± 2
S-2	square prism	40	40	0.5	0.250	1.800	155 ± 1	164 ± 1	152 ± 2	12 ± 2
S-3	cylinder	40	40	0.5	0.196	1.628	152 ± 2	161 ± 1	152 ± 2	9 ± 2

The Cassie–Baxter solid fraction, *f*_s – sq_^CB^ and linear lubricant fraction, *l*_f_, of the S-1 and S-2 square micropatterned samples were selected to be identical with two square pattern samples given in ref (62) (*f*_s – sq_^CB^= 0.16, *l*_f_ = 0.6 and *f*_s – sq_^CB^= 0.25, *l*_f_ = 0.5 respectively), as seen in Table 1. We also selected *d*, *s*, and *l*_f_ values of the S-3 sample equal to the S-2 sample for better comparison of the results between square and cylindrical micropatterned SLIPS samples.

Silicone oil generally spreads on flat, smooth hydrophobized surfaces under air, impregnates, and flows atop pillars by the action of capillarity forces.^32−34,62^ When a water drop is placed on a SLIP surface, there may be two possibilities: the drop may contact with the top of the solid pillars by displacing the lubricant layer or may rest on the liquid lubricant film over the pillars without contacting the solid. It is possible to distinguish between these two cases easily since the evaporation of the water drop in the former case would not follow the CCA mode by showing a stick–slip behavior while it follows the CCA mode in the latter case.^62^ For the possible industrial applications of SLIPS, the presence of the latter case is more important, and the optimization of the thickness and viscosity of the lubricant layer is required for low operational costs. The effect of pattern geometry and the corresponding ridge height is crucial for both water vapor condensation and drop evaporation applications of SLIP surfaces.

The water contact angles on the flat DMDCS-coated silicon wafer were measured to be θ_app_ = 105 ± 1°, θ_a_ = 106 ± 1°, θ_r_ = 102 ± 2°, and CAH = 4 ± 2°. As seen in Table 1, all the water contact angles (θ_app_, θ_a_, θ_r_, and CAH values) on the DMDCS-coated pillar patterns were very close to each other for the superhydrophobic S-1 and S-2 samples made of square prism pillars. However, there was a slight decrease in all these values for the S-3 samples, where cylindrical pillars were used. This was due to the presence of curvatures on the top of the cylindrical pillars instead of sharp edges.^81^ The silicone oil contact angles on the DMDCS-coated silicon wafer were measured to be θ_app_ = 7 ± 1° for the 20 cSt-type and θ_app_ = 17 ± 1° for the 350 cSt-type. The silicone oil contact angles on the hydrophobized flat substrates were very low and much smaller than the critical contact angle (θ_critical_) values of 76–82°, which were calculated as defined by Quere and co-workers.^32,33^ Thus, the impregnation of the silicone oil lubricant and covering the top of the hydrophobized pillars of the patterns by the action of capillarity forces happened for all the SLIPS samples.

The stability of the silicone oil layer on the DMDCS-coated patterns under water drops were also examined. A stable silicone oil layer on a patterned substrate can be formed rather than a water layer if the following criteria, [*r*_w_(γ_OV_ cos θ_O_ – γ_LV_ cos θ_L_) – γ_OL_ > 0] and [*r*_w_(γ_OV_ cos θ_O_ – γ_LV_ cos θ_L_) + γ_LV_ – γ_OV_ > 0], are being satisfied.^34,68^*r*_w_ is the Wenzel roughness factor, γ_OV_ is the oil–air surface tension, γ_LV_ is the water–air surface tension, γ_OL_ is the oil–water interfacial tension, and θ_L_ and θ_O_ are the apparent contact angles of water and silicone oils on the flat DMDCS substrate, respectively. *r*_w_ varies between 1.628 and 2.024 for our three patterns as given in Table 1, θ_L_ = 105 ± 1°, θ_O_ = 7 ± 1° for 20 cSt silicone oil, and θ_O_ = 17 ± 1° for 350 cSt silicone oil. γ_LV_ = 72.8 mN/m,^67^ γ_OV_ = 21.0 mN/m,^67^ and γ_OL_ = 39.5 mN/m.^67^ By using these values, it was calculated that, *r*_w_(γ_OV_ cos θ_O_ – γ_LV_ cos θ_L_) – γ_OL_ > 0 since it varies between 23.87 and 40.82 mN/m. Similarly, *r*_w_(γ_OV_ cos θ_O_ – γ_LV_ cos θ_L_) + γ_LV_ – γ_OV_ > 0 since it varies between 115.17 and 132.12 mN/m, indicating that a stable silicone oil layer on the patterned substrates could be formed rather than a water layer on all of the DMDCS-coated samples coated by both 20 and 350 cSt silicone oils.

### Increase of Ridge Height on the Water Drop with the Increase of the Height of the Silicone Oil Lubricant

After filling the empty volume of the hydrophobized silicone patterns with silicone oil, which was calculated from the geometry of the pillars, more silicone oil was added incrementally to cover the tops of the pillars with a lubricant film and the initial heights of the silicone oil (*h*_oil_)_i_ over the pillars were calculated gravimetrically using the added oil masses divided by the total area of the samples (400 mm^2^). After placing the water drops, the initial ridge heights (*h*_r_)_i_ on the water drops were measured as given above, and the results are given in Table S1 in the Supporting Information file.

As seen in Table 1 and Table S1, there was an inverse relationship with the Wenzel roughness factor (*r*_w_) and initial ridge heights (*h*_r_)_i_ around the water drop, although its linearity was poor. The increase in *r*_w_ indicates an increase of the oil–solid contact area, and a decrease in (*h*_r_)_i_ around the water drop can be explained with the decreased availability of the required silicone oil molecules to fill the skirt around the drop due to larger oil–solid interfacial interactions. On the other hand, there was no meaningful relationship of (*h*_r_)_i_ with (*f*_s_^CB^) and (*l*_f_).

**Figure 5:**
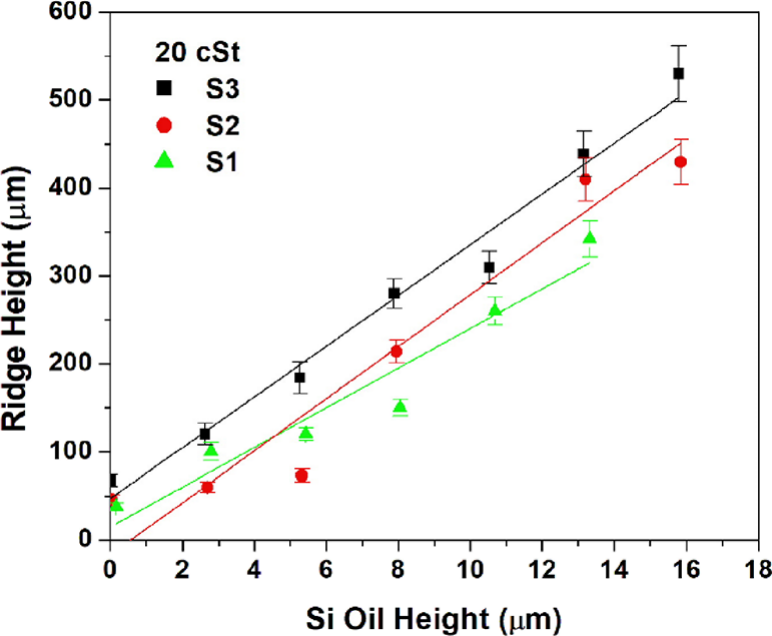
Change of initial ridge height (*h*_r_)_i_ with the change in initial silicone oil height (*h*_oil_)_i_ on SLIPS for lubricant oil with 20 cSt viscosity.

**Figure 6:**
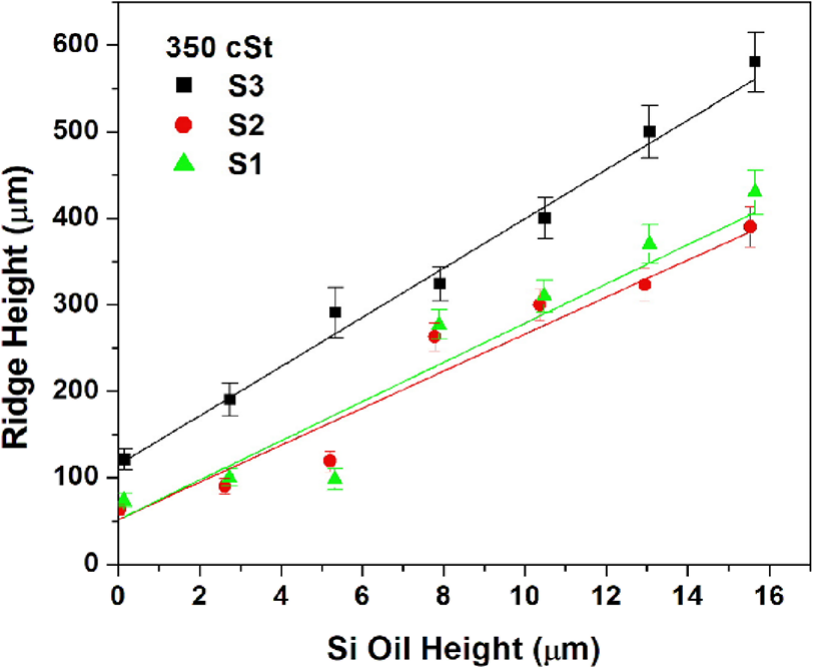
Change of initial ridge height (*h*_r_)_i_ with the change in initial silicone oil height (*h*_oil_)_i_ on SLIPS for lubricant oil with 350 cSt viscosity.

The effect of the initial lubricant oil heights (*h*_oil_)_i_ on the initial ridge heights (*h*_r_)_i_ can be seen when the data in Table S1 is plotted in Figures 5 and 6 with error bars, where the (*h*_r_)_i_ values increased almost linearly with the increase of (*h*_oil_)_i_ for all cases. When the effect of the pattern geometry is considered, the (*h*_r_)_i_ values were largest on the SLIPS samples made of cylindrical pillars (S-3) for all the (*h*_oil_)_i_ values. Very close (*h*_r_)_i_ values were obtained for the square prism-patterned S-1 and S-2 samples especially when 350 cSt silicone oil was used as a lubricant. It is possible that the SLIPS samples made of patterns with cylindrical pillars had a higher wetting ridge for the same oil thickness due to the presence of more “reservoir” space in between the pillars, which allows more lubricant to be pulled upward.

When the effect of the kinematic viscosity of silicone oil on both (*h*_oil_)_i_ and (*h*_r_)_i_ is considered, the rise of lubricant viscosity from 20 to 350 cSt caused an increase in (*h*_r_)_i_ values especially for low (*h*_oil_)_i_ values. With the increase of the oil heights, the lubricant viscosity effect on the (*h*_r_)_i_ parameter was found to be slight, especially for the S-3 samples with cylindrical pillars, after the (*h*_oil_)_i_ value approached 8 μm. The linearity was found to be best on the S-3 SLIPS samples in both Figures 5 and 6.

### Evaporation Rates of Water Drops on the SLIP Surfaces

**Figure 7:**
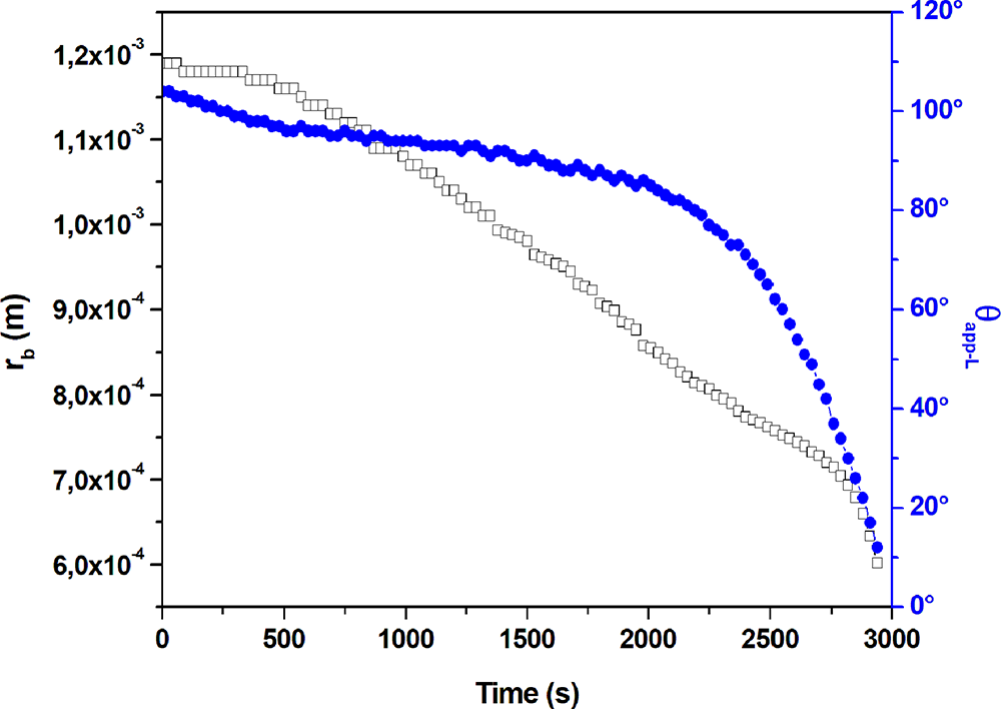
Change of contact radius (*r*_b_) and apparent contact angle at the liquid three-phase contact line at the lubricant–water–air phases (θ_app-L_) during the evaporation of a water drop placed on the SLIPS sample formed by infusing 20 cSt silicone oil onto the S-1 pattern with an initial top oil layer thickness (*h*_oil_)_i_ of 0.16 μm and initial ridge height (*h*_r_)_i_ of 33 μm.

**Figure 8:**
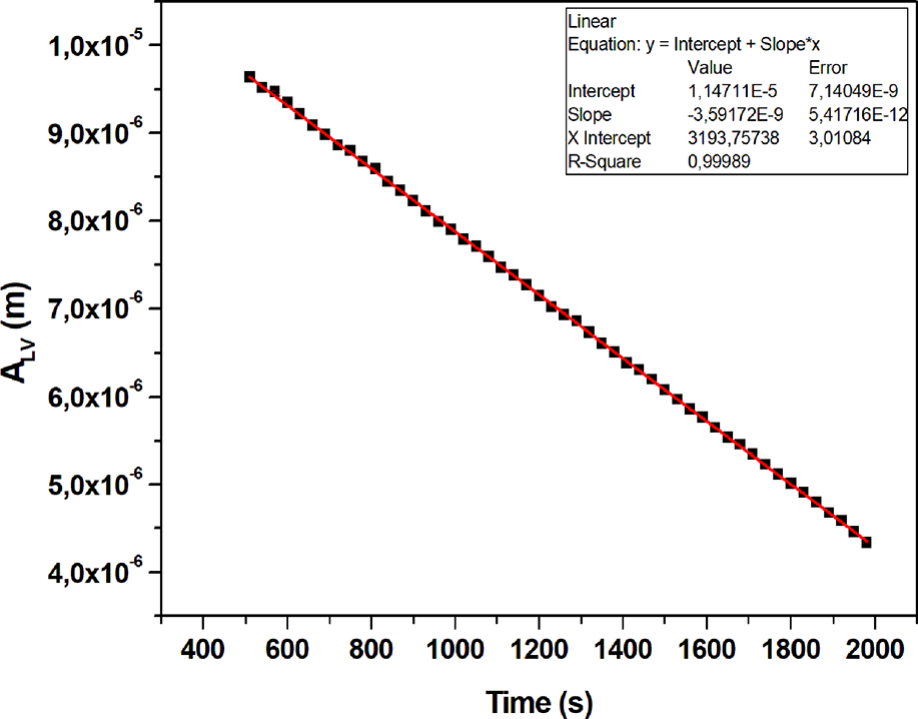
Change of liquid–air interfacial area of water drop (*A*_LV_) during the evaporation of a water drop on a SLIPS sample formed by infusing 20 cSt silicone oil on the S-1 pattern with an initial top oil layer thickness (*h*_oil_)_i_ of 0.16 μm and initial ridge height (*h*_r_)_i_ of 33 μm.

The results of the change of drop contact radius (*r*_b_) and apparent contact angle (θ_app-L_) with time during the evaporation of water drops from the SLIPS samples are given in Figures 7 and 8 and Figures S1 to S45 in the Supporting Information file. All experiments were carried out at a constant RH of 54 ± 1% and at 25 ± 1 °C temperature in a transparent plexiglass closed cell. A total of 24 × 3 = 72 drop evaporation experiments from the SLIPS samples were implemented for two different silicone oil viscosities (20 and 350 cSt) on three different samples (S-1, S-2, S-3) by forming four different silicone oil layer thicknesses.

The objective in these experiments is the determination of the effect of initial silicone oil film heights above the substrate pattern (*h*_oil_)_i_ and initial lubricant oil ridge heights around the water drop (*h*_r_)_i_ on the drop evaporation rates and lifetimes of the water drops. The drop evaporation results from SLIP surfaces at constant 25 °C and 55% RH are given in Table 2. Experimental values of (*h*_oil_)_i_ and (*h*_r_)_i_ and corresponding initial θ_app-L_ and θ_app-S_ are presented with the lifetimes of the drops having the same initial volume of 5 μL of pure water. (d*A*_LV_/d*t*) values are taken from the slopes of plots of the free interfacial area decrease of the three-dimensional spherical liquid drops at the liquid/vapor interface (*A*_LV_) with time as given in Figure 8 and Figures S2, S4, S6, S8, S10, S12, S14, S16, S18, S20, S22, S24, S26, S28, S30, S32, S34, S36, S38, S40, S42, S44, and S46 in the Supporting Information file.

**Table 2:** Experimental Drop Evaporation Results from SLIP Surfaces at 25 °C (298.15 K) Temperature and 55% RH Where Saturated Water Vapor Pressure Was *P*_v_^s^ = 3169 Pa, Diffusion Constant of Water Vapor in Air Was *D*_literature_ = 2.60 × 10^–5^ m^2^/s, and *M*_w_(H_2_O) = 0.01801524 kg/mol^a^

	sample	initial (*h_r_*)_*i*_ (μm)	initial (*h_oil_*)_*i*_ (μm)	θ_app-L_ (^o^)	θ_app-S_ (^o^)	*f*(θ_app-L_)	(d*A_LV_*/d*t*) 10^–9^ (m^2^/s)	lifetime (s)	*D*-Exp 10^–5^ (m^2^/s)	error %
20 cSt Si oil	S-1	33	0.16	93	96	0.513	3.592	2940	2.80	7.7
		119	5.47	95	98	0.521	3.184	3500	2.57	1.1
		146	8.06	91	101	0.504	3.115	3500	2.55	2.0
		333	13.30	63	98	0.367	1.866	4800	2.06	20.6
	S-2	46	0.05	88	91	0.491	3.423	3060	2.69	3.5
		73	5.32	89	95	0.496	3.157	3120	2.58	0.9
		193	7.90	80	88	0.454	3.043	3450	2.52	3.1
		448	13.21	65	95	0.378	1.820	4680	1.92	26.0
	S-3	66	0.01	99	104	0.537	3.248	3600	2.53	2.9
		184	5.26	89	100	0.496	2.770	3840	2.29	11.8
		280	7.87	62	94	0.361	2.020	4380	2.21	15.0
		448	13.27	58	92	0.339	1.753	4410	2.01	22.67
350 cSt Si oil	S-1	74	0.14	93	97	0.513	3.361	3600	2.67	2.6
		129	5.35	87	91	0.487	2.921	3960	2.22	14.6
		277	7.89	83	99	0.468	2.145	4560	2.27	12.5
		310	13.06	65	91	0.378	1.856	4860	1.90	27.0
	S-2	37	0.03	97	101	0.529	3.143	3600	2.45	5.8
		67	3.20	89	98	0.496	2.873	3570	2.35	9.5
		233	7.75	80	98	0.454	2.359	4680	2.11	18.9
		328	12.96	62	97	0.361	1.892	4710	2.12	18.8
	S-3	124	0.16	92	99	0.509	3.004	3720	2.41	7.3
		285	5.31	81	96	0.459	2.409	4530	2.10	19.1
		344	7.92	64	97	0.372	2.076	4560	2.25	13.5
		398	11.40	71	96	0.409	2.023	4530	1.98	23.8

a The error % given in the far right column depicts the deviation of the experimentally determined diffusion constant of water vapor in air (*D*-Exp) from the *D*literature value.

The linearity of the (d*A*_LV_/d*t*) plots was good during the CCA mode of drop evaporation for θ_app-L_. However, in most of the experiments, the CCA mode starts after a while, ranging between 200 and 700 s, and in some instances, a mean value of θ_app-L_ was used for the constant contact angle since a simultaneous and slow decrease was seen for both θ_app-L_ and *r*_b_ for some plots. The slopes of the (d*A*_LV_/d*t*) plots indicate the drop evaporation rates. The diffusion constants for water vapor in air (*D*) were calculated by using the slopes of the (d*A*_LV_/d*t*) plots in eq 16, and the *D* error percentages were found by comparing with the value reported in the literature, *D*_literature_ = 2.60 ×10^–5^ m^2^/s,^82^ and given in Table 2.

**Figure 9:**
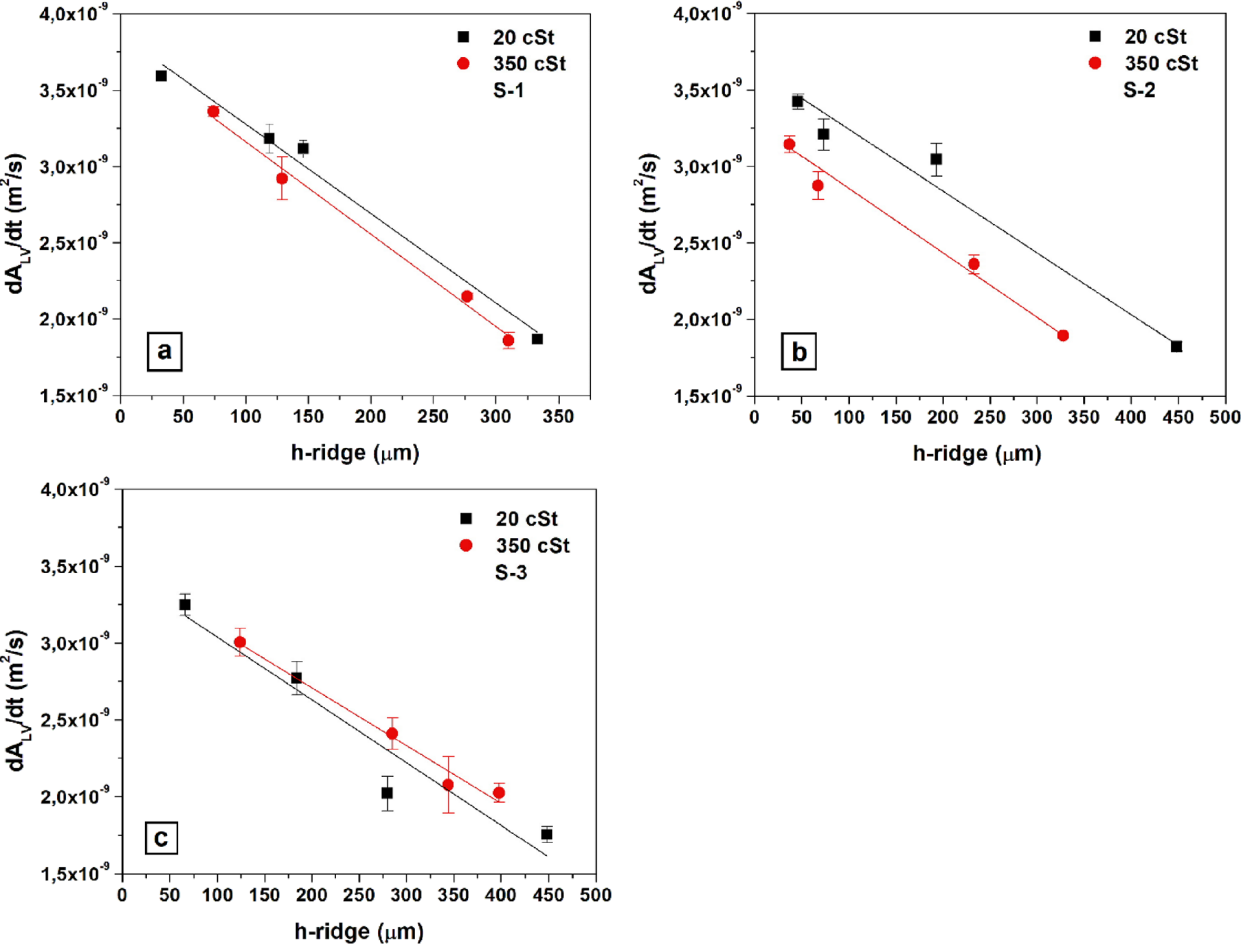
Change of drop evaporation rate via the change of liquid–air interfacial area of water drop by time (d*A*_LV_/d*t*) with the change in the initial oil ridge heights (*h*_r_)_i_ on SLIPS samples that were formed by infusing with 20 and 350 cSt silicone oils: (a) S-1 sample, (b) S-2 sample, and (c) S-3 sample.

**Figure 10:**
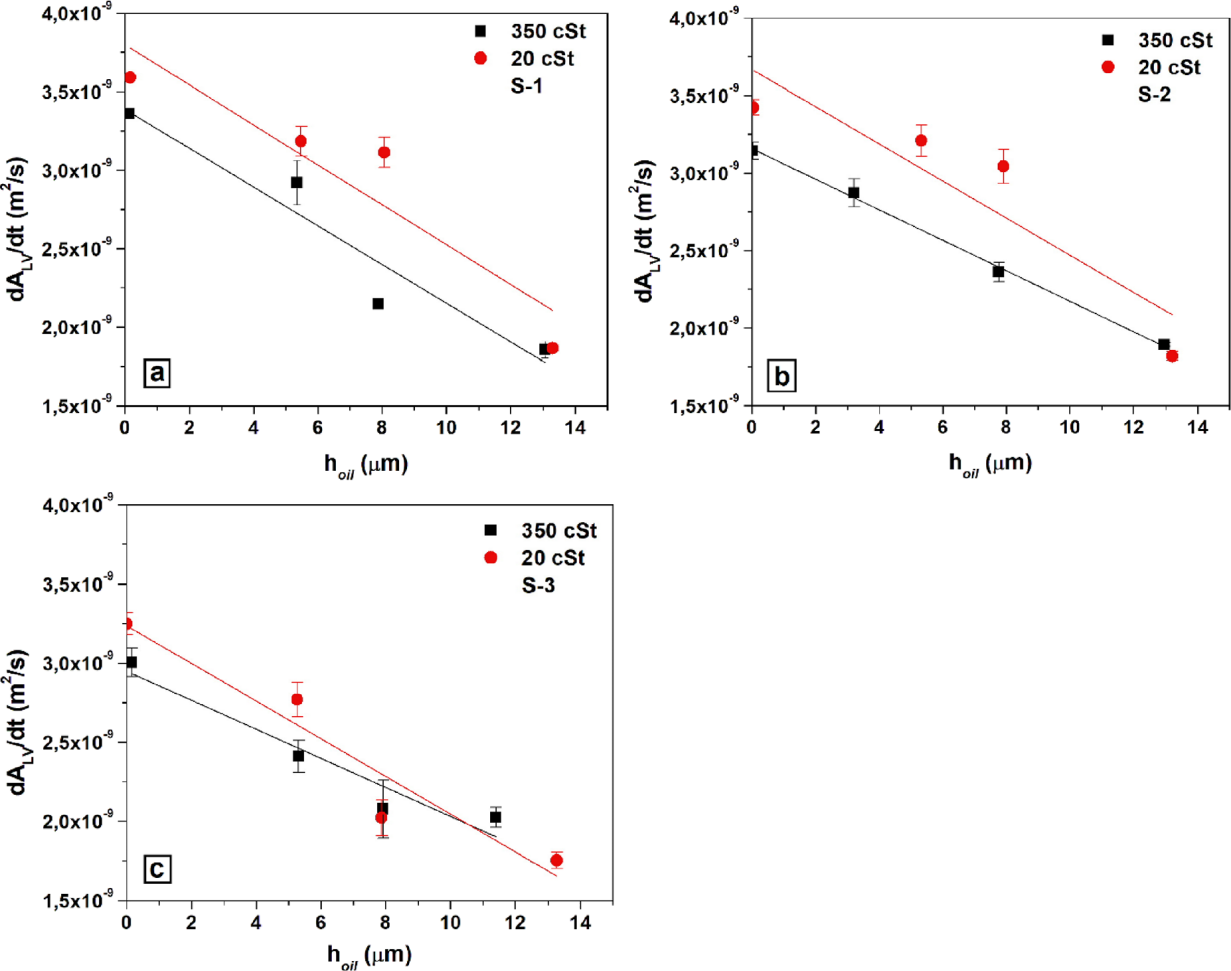
Change of drop evaporation rate via the change of liquid–air interfacial area of the water drops by time (d*A*_LV_/d*t*) with the change in the initial oil film heights above the substrate patterns (*h*_oil_)_i_ on the SLIPS samples, which were formed by infusing with 20 and 350 cSt silicone oils: (a) S-1 sample, (b) S-2 sample, and (c) S-3 sample.

The change of (d*A*_LV_/d*t*) values with the change of initial ridge heights (*h*_r_)_i_ as well as the initial oil heights (*h*_oil_)_i_ would guide us in controlling the drop evaporation (as well as the drop condensation) rates. The decrease of (d*A*_LV_/d*t*) values with the increase of (*h*_r_)_i_ on the S-1, S-2, and S-3 SLIPS samples, which are infused by both 20 and 350 cSt silicone oil lubricants, are given in Figure 9 with error bars. Similarly, the decrease of (d*A*_LV_/d*t*) values with the increase of (*h*_oil_)_i_ on the S-1, S-2, and S-3 SLIPS samples infused by 20 and 350 cSt silicone oil lubricants are given in Figure 10 with error bars.

As seen in Table 2 and Figure 9, there was a linear inverse relationship between (*h*_r_)_i_ and (d*A*_LV_/d*t*) values for all the drop evaporation results. This behavior is expected since the available total liquid/air interfacial area decreased with the increase of (*h*_r_)_i_. Meanwhile, the initial θ_app-L_ values were found to decrease slightly with the increase of both (*h*_oil_)_i_ and (*h*_r_)_i_; however, the effects of (*h*_oil_)_i_ and (*h*_r_)_i_ on the initial θ_app-S_ values were negligible, similar to the previous reports^62,78,84,85^ and indicating that the drops retained the shape of their profiles without any (*h*_oil_)_i_ effect at the horizontal lubricant level just above the substrate at the intersection between the infusing liquid, water, and the solid. On the other hand, the θ_app-L_ values decreased with the increase of (*h*_oil_)_i_, and if the (*h*_oil_)_i_ was larger than a threshold value of 8 μm, then the initial θ_app-L_ decreased sharply due to the better supply of silicone oil molecules from the thick oil layers to the three-phase contact lines of oil–water drop–air. In general, the increase of *h*_oil_ caused a decrease in the initial water droplet height and corresponding initial *A*_LV_ value and increased in drop lifetimes similar to previous reports.^67^ On the other hand, the effect of changing the viscosity of the infused silicone oil on the initial θ_app-L_ and θ_app-S_ values was small depending on the used pattern type. For SLIPS samples infused by 20 cSt silicone oil, the higher initial θ_app-L_ and θ_app-S_ values were obtained on the S-3 samples, which were made of cylindrical pillars, compared to the S-2 and S-3 samples with square prism pillars. Meanwhile, for the SLIPS samples infused by 350 cSt silicone oil, the initial θ_app-L_ and θ_app-S_ values were close to each other (deviates less than ±4%) for all the patterns.

The effect of low-viscosity (20 cSt) silicone oil giving higher initial θ_app-L_ and θ_app-S_ values on the cylindrical S-3 SLIPS sample may be due to the presence of curved cylindrical pillars, which causes the rapid transport of silicone oil molecules into the ridge volume in comparison with the less viscous case.

On the other hand, the effect of the initial ridge heights (*h*_r_)_i_ on the drop evaporation rates was considerable on all the SLIPS samples. As seen in Figure 9a, the linear decrease of drop evaporation rates (d*A*_LV_/d*t*) with the increase of (*h*_r_)_i_ were close to each other for both 20 and 350 cSt lubricant oil viscosities for the S-1 SLIPS samples made of small square prism pillars with regression coefficients of *R*^2^ = 0.988–0.993. The numerical values of (d*A*_LV_/d*t*) on the 20 cSt oil-infused samples were around 8% higher (mean values) than that of the 350 cSt-infused samples for similar ridge heights. Correspondingly, the lifetimes of evaporating water drops on the 350 cSt oil-infused samples were around 12% higher (mean values) than that of the 20 cSt-infused samples. In summary, the increase of the infused lubricant viscosity caused a decrease in the drop evaporation rates and an increase in the drop lifetimes.

The calculation of the diffusion constant, *D*, of water vapor in air from (d*A*_LV_/d*t*) values by using eq 16 was considerably successful for (*h*_oil_)_i_ values up to 8 μm within ±7% on the S-1 SLIPS samples. However, when the (*h*_oil_)_i_ level was above 8 μm and (*h*_r_)_i_ > 146 μm, large deviations (13–27%) were seen from the *D*_literature_ = 2.60 × 10^–5^ m^2^/s value due to the possible formation of a thin silicone oil cloaking layer on the drop surface, which prevented water evaporation considerably. All of the deviations in *D* values were due to the slow evaporation rates of the water drops when the (*h*_oil_)_i_ was large. Similarly, the magnitudes of (*h*_oil_)_i_ and (*h*_r_)_i_ had an effect on the initial θ_app-L_ values after a threshold value. The initial θ_app-L_ values were measured to be between 91 and 95° for 20 cSt and 83 and 93° for 350 cSt-infused samples up to a (*h*_oil_)_i_ value of 8 μm and then decreased down to 63–65° with the increase in both (*h*_oil_)_i_ and (*h*_r_)_i_ values. However, there were negligible effects of (*h*_oil_)_i_ and (*h*_r_)_i_ values on the initial θ_app-S_, which were measured to be between 96 and 101° for 20 cSt and 91 and 97° for the 350 cSt-infused samples, similar to the previous reports.^62,78,84,85^

For the S-2 SLIPS samples where large square prism pillars were used as the substrate pattern, the decrease of drop evaporation rates (d*A*_LV_/d*t*) with the increase of (*h*_r_)_i_ were linear with regression coefficients of *R*^2^ = 0.968–0.983 as seen in Figure 9b, similar to the results determined on the S-1 samples having patterns with small square prism pillars. The numerical values of (d*A*_LV_/d*t*) on the 20 cSt oil-infused SLIPS samples were around 12% higher (mean values) than that of the 350 cSt-infused samples. The lifetimes of the evaporating water drops on the 350 cSt oil-infused samples were around 17% higher (mean values) than that of the 20 cSt-infused samples. The calculation of *D* from (d*A*_LV_/d*t*) values using eq 16 was considerably good for the (*h*_oil_)_i_ values up to 8 μm within ±7% on the S-2 SLIPS samples. However, the increase of the (*h*_oil_)_i_ level above 8 μm and (*h*_r_)_i_ above 193 μm caused large deviations (18.8–26.0%) from the *D*_literature_ value due to the possible presence of a thin silicone oil cloaking layer blocking the water drop surface and resulted in slow evaporation. The initial θ_app-L_ values were found to be between 80 and 88° for 20 cSt and 80 and 97° for the 350 cSt-infused samples up to 8 μm (*h*_oil_)_i_ and decreased down to 62–65° with the increase in (*h*_oil_)_i_ and (*h*_r_)_i_ values. There was a negligible effect of (*h*_oil_)_i_ and (*h*_r_)_i_ values on the initial θ_app-S_, which was varied between 88 and 95° for 20 cSt and 98 and 101° for the 350 cSt-infused samples.

For the S-3 SLIPS samples where cylindrical pillar patterns were used as the substrate, the linear decrease of drop evaporation rates (d*A*_LV_/d*t*) with the increase of (*h*_r_)_i_ were close to each other for both 20 and 350 cSt lubricant oil viscosities with *R*^2^ regression coefficients of 0.923–0.980, as seen in Figure 9c. However, the linearity of the plots that was found on the S-3 SLIPS samples was smaller than the linearity of the plots on S-1 and S-2 samples, as can be seen in the regression coefficient values. Moreover, a reverse trend was seen on the S-3 samples where the numerical values of (d*A*_LV_/d*t*) on the 350 cSt oil-infused samples were around 8% higher (mean values) than that of the 20 cSt-infused samples for large (*h*_oil_)_i_ and (*h*_r_)_i_ values. The lifetimes of evaporating water drops on the 350 cSt oil-infused samples were around 7% higher (mean values) than that of the 20 cSt-infused samples. The calculation of *D* of water vapor in air from (d*A*_LV_/d*t*) values using eq 16 was satisfactory for the initial oil heights up to 8 μm values within ±12% on the S-3 SLIPS samples. The increase of (*h*_oil_)_i_ above 8 μm and (*h*_r_)_i_ above 344 μm caused large deviations (13.5–23.8%) from the *D*_literature_ value due to the formation of a thin silicone oil cloaking layer, giving slow evaporation rates. The initial θ_app-L_ values were found to be between 89 and 99° for 20 cSt and 81 and 92° for 350 cSt-infused samples up to 5 μm (*h*_oil_)_i_ and decreased down to 58–71° with the increase in (*h*_oil_)_i_ and (*h*_r_)_i_ values. There was only a small effect of (*h*_oil_)_i_ and (*h*_r_)_i_ values on the initial θ_app-S_, which were varied between 92 and 104° for 20 cSt and 96 and 99° for the 350 cSt-infused samples.

In summary, it was determined that the magnitude of (*h*_oil_)_i_ on the water drop evaporation rates was important on all the SLIPS samples. As seen in Table 2 and Figure 10, the increase in (*h*_oil_)_i_ decreased the evaporation rates (d*A*_LV_/d*t* values) roughly linearly for all the drop evaporation experiments. This is expected since the corresponding (*h*_r_)_i_ is proportional with the (*h*_oil_)_i_ value as given in Figures 5 and 6. In general, the effect of (*h*_oil_)_i_ on the drop evaporation rates of the water drops placed on the S-1, S-2, and S-3 SLIPS samples is similar to the effect of (*h*_r_)_i_ on the drop evaporation rates, but there is less linearity since the *R*^2^ regression coefficients varied between 0.831 and 0.997 in comparison with 0.923–0.993. For the case of the pattern effect, it was found that drop evaporation rates were slightly higher on patterns made of small square pillars than that of the large square pillars and there were very small effects of the type of pillar (square or cylindrical) on the drop lifetimes since the lubricant oil covered the pillar tops.

The practical importance of the relation of (*h*_oil_)_i_ with the drop evaporation rate is the prediction of the drop lifetimes according to the supplied lubricant oil thickness. This result is also important for the drop condensation studies on SLIP surfaces, which is a crucial topic in industrial heat transfer applications since it is possible to use the diffusion-controlled drop evaporation expressions in drop condensation studies in many instances.^83^

It was also determined that the time-dependent *h*_r_ values can be higher than the (*h*_r_)_i_ values, especially during the half-lifetime of the drop evaporation experiments when 20 cSt silicone oil was used as lubricant, as given in Table S2 in the Supporting Information file. However, this condition was not valid for the experiments when 350 cSt silicone oil was used, where the (*h*_r_)_i_ values were nearly constant during the half-drop lifetime due to the limited oil supply from the viscous silicone oil.^66^ It was seen that the *h*_r_ started to diminish along with the drop height until the end of the evaporation, similar to the results reported in ref (67). The growth of *h*_r_ is limited by the flow from the lubricant around the drop, and the equilibrium value of *h*_r_ may be achieved after a long time since it corresponds to the value where the radius of curvature of the wetting ridge becomes roughly equal to the drop radius or capillary length of the lubricating fluid.^85^

We determined that the increase of (*h*_oil_)_i_ above 8 μm resulted in large deviations (13–27%) from the *D*_literature_ = 2.60 × 10^–5^ m^2^/s value on all SLIPS samples, possibly due to the formation of a silicone oil cloaking layer on the drop surface, which prevented water evaporation and gave low (d*A*_LV_/d*t*) values. There is a similar report in the literature that the amount of oil available for drop cloaking increases with the rise in oil layer thickness.^68^ It is known that lubricant cloaking occurs, which is the formation of a wrapping layer of lubricant over the water drops when the spreading coefficient of lubricant over the water droplet is positive, that is, *S*_OL(V)_, the spreading coefficient of oil on water under air (*S*_OL(V)_ = γ_LV_ – γ_OL_ – γ_OV_) >0, where γ_LV_, γ_OV_, and γ_OL_ are the interfacial energies of the water–air, lubricant–air, and lubricant–water interfaces, respectively.^34,62,84−86^ By using the values given above—γ_LV_ = 72.8 mN/m,^67^ γ_OL_ = 39.5 mN/m,^67^ and γ_OV_ = 21.0 mN/m^67^—we obtained a positive value of *S*_OL(V)_ = 12.3 mN/m, indicating that both of the 20 and 350 cSt silicone oils should cloak the droplet, but we did not directly observe such an effect in our drop evaporation experiments until the thickness of the initial silicone oils on the patterns exceeded 8 μm. It is probable that the oil layer either did not wrap the water drop completely or was sufficiently thin with negligible effect on the drop evaporation rates.^62^ However, after adding (*h*_oil_)_i_ > 8 μm on the solid patterns, the rate of drop evaporation decreased considerably, giving large deviations from the constant *D* values. Thus, we need to calculate the equilibrium lubricant film thickness above the water drop (*h*_w_) according to the procedure given in refs (84). and (85). The *h*_w_ can be found by equating the capillary pressure in the cloaking film caused by the drop curvature, (2γ_OV_/*R*), where *R* is the radius of curvature of the drop, and the disjoining pressure, Π_(h)_. The wetting ridge is assumed to be a low-pressure region, and lubricant will drain out from the cloaking layer to the wetting ridge until it reaches a nanometric equilibrium thickness, where it is stabilized by van der Waals forces, leading to a repulsive disjoining pressure between the water–oil and oil–air interfaces. For this condition, the Hamaker constant, *A*_H_, and the resultant disjoining pressure, Π_(*h*)_ = *A*_H_/6π*h*_w_^3^, in the lubricant film should be positive.^84−86^*A*_H_ quantifies the strength of the interactions between the molecules of the contacting phases. The film thickness of the cloaking layer can be found using
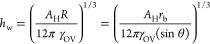
17

Assuming the Hamaker constant value for the air–water–silicone oil interface to be 4 × 10^–21^ J^68^ and using the average measured initial contact radius of all drops, *r*_b_ = 1.28 × 10^–3^ m, and average contact angle of θ_app-S_ = 95°, the equilibrium cloaking oil film thickness on a water drop is calculated to be *h*_w_ ≈ 19 nm similar to the values reported in refs (68) and (84). The effect of interfacial van der Waals forces is significant within the cloaking layer since the *h*_w_ is much lower than 100 nm. Thus, more experimental works are required on the thickness of the cloaking lubricant layers with the increase of (*h*_oil_)_i_ > 8 μm since it may also be possible that the silicone oil film only wraps around the lower side of a water drop to form an extended thick skirt ridge just above the wetting ridge and constitutes a much thinner layer toward the top of the droplet.

## Conclusions

It was determined that the control on the initial lubricant silicone oil heights (*h*_oil_)_i_ and corresponding initial ridge heights around the water drops (*h*_r_)_i_ can guide us on how to conduct the drop evaporation rates on SLIP surfaces. The (*h*_r_)_i_ increased almost linearly on the lower parts of the water drops with the increase of (*h*_oil_)_i_ on the pattern surfaces for all cases. When the effect of the substrate pattern geometry was considered, the (*h*_r_)_i_ values were largest on the SLIPS samples made of cylindrical pillars (S-3), and close (*h*_r_)_i_ values were obtained for S-1 and S-2 samples made of square prism pillars, especially when 350 cSt silicone oil was used as lubricant, indicating that the effect of the size of the square prism pillars was negligible. A non-linear inverse relationship was found between the Wenzel roughness factor (*r*_w_) of the solid pattern and (*h*_r_)_i_, showing the importance of solid/oil contact area. The effect of the silicone oil kinematic viscosity on the drop evaporation rates was small and disappeared with the increase of (*h*_oil_)_i_. Similarly, the variation of the infused silicone oil viscosity did not affect both the initial θ_app-L_ and θ_app-S_ values. The effect of (*h*_oil_)_i_ on the apparent contact angles of the water drops was minimal up to a threshold value of (*h*_oil_)_i_ = 8 μm, where the initial θ_app-L_ decreased slightly with the increase of (*h*_oil_)_i_ and then decreased sharply after the threshold (*h*_oil_)_i_ value. Moreover, there was a very small effect of (*h*_oil_)_i_ and (*h*_r_)_i_ values on the initial θ_app-S_. The effect of pattern geometry on the apparent contact angles on SLIPS was small, and higher initial θ_app-L_ values were obtained on the S-3 samples, which were made of cylindrical pillars, when they were infused by 20 cSt silicone oil, but this condition was not valid when the S-3 samples were infused by 350 cSt oil.

A novel equivalent diffusion-limited drop evaporation equation from SLIPS (eq 16) was derived depending on the available free liquid–air interfacial area. This expression is better to describe the drop evaporation process on SLIPS since it contains the free liquid–air interfacial area parameter, *A*_LV_, which directly shows the unblocked part of the total drop surface with the lubricant skirt. A linear inverse relationship was found between the (*h*_r_)_i_ and (d*A*_LV_/d*t*) values for all the drop evaporation results, in accordance with the decrease of total liquid/air interfacial area with the increase of (*h*_r_)_i_. Similarly, the increase in the (*h*_oil_)_i_ values decreased the evaporation rates (d*A*_LV_/d*t*) less linearly for all the drop evaporation experiments since (*h*_r_)_i_ was proportional to (*h*_oil_)_i_. Thus, it is possible to control the drop evaporation rates by adjusting the lubricant thickness over the solid patterns. Meanwhile, the increase of the infused lubricant silicone oil viscosity caused only a slight decrease (8–12%) in the drop evaporation rates and a small increase of 12–17% in the drop lifetimes especially on patterns having square prism pillars. For the effect of the type and size of pillars, only minimal variations were found and the drop evaporation rates increased slightly on SLIPS with patterns made of smaller pillars and cylindrical types for the conditions where the silicone oil lubricant covered the pillar tops.

The diffusion constant, *D*, of water vapor in air from (d*A*_LV_/d*t*) values were successfully calculated by using eq 16 up to a threshold value of (*h*_oil_)_i_ = 8 μm within ±7% on SLIPS samples, and the increase of (*h*_oil_)_i_ above this value resulted in large deviations (13–27%) of *D*, possibly due to the formation of a thin silicone oil cloaking layer on the drop surface that blocked the drop evaporation.

## Supporting Information Available

Table for the change of the initial ridge height (*h*_r_)_i_ around the water drop with the increase of initial silicone oil height (*h*_oil_)_i_ on the micropatterned samples; table for the change of initial ridge height (*h*_r_)_i_ around the water drop after half-lifetime during evaporation on the micropatterned SLIPS samples infused by 20 cSt silicone oil; plots for the change of the contact radius (*r*_b_) and apparent contact angle at the liquid three-phase contact line at the lubricant–water–air phases (θ_app-L_) during the evaporation of water drops placed on the SLIPS samples formed by infusing 20 and 230 cSt silicone oil onto the S-1, S-2, and S-3 patterns with varying initial top oil layer thicknesses (*h*_oil_)_i_ and initial ridge heights (*h*_r_)_i_; plots for the change of the liquid–air interfacial area of the drops (*A*_LV_) during the evaporation of water drops on SLIPS samples formed by infusing 20 and 350 cSt silicone oil on S-1, S-2, and S-3 patterns with varying (*h*_oil_)_i_ and (*h*_r_)_i_ values (PDF)
