# Supplementary material for: Water Drop Evaporation on Slippery Liquid-Infused Porous Surfaces (SLIPS): Effect of Lubricant Thickness, Viscosity, Ridge Height, and Pattern Geometry
Source: Langmuir. 2023 Apr 27;39(18):6514–28. doi: 10.1021/acs.langmuir.3c00471 (PMC10173461; doi:10.1021/acs.langmuir.3c00471)
Supplement: Supplementary file 1 — la3c00471_si_001.pdf [file la3c00471_si_001.pdf]

Supporting Information:

Water Drop Evaporation on Slippery Liquid-Infused Porous Surfaces (SLIPS): Effect of Lubricant Thickness, Viscosity, Ridge Height, and Pattern Geometry

Rana Üçüncüoğlu, H. Yildirim Erbil*

Department of Chemical Engineering, Gebze Technical University, Gebze 41400, Kocaeli, Türkiye.

Table of Contents of Supporting Information:

Number	Caption	Page
Table S.1	The change of the initial ridge height $(h_r)_i$ around the water drop with the increase of initial silicone oil height $(h_{oil})_i$ on the micropatterned samples.	2
Table S.2	The change of initial ridge height $(h_r)_i$ around the water drop after half life-time during evaporation on the micropatterned SLIPS samples infused by 20 cSt silicone oil.	2
Figures S.1, S.3, S.5, S.7, S.9, S.11, S.13, S.15, S.17, S.19, S.21, S.23, S.25, S.27, S.29, S.31, S.33, S.35, S.37, S.39, S.41, S.43, S.45	Plots for the change of contact radius (r_b) and apparent contact angle at the liquid three-phase contact line at the lubricant-water-air phases (θ_{app-L}) during the evaporation of water drops placed on SLIPS samples which were formed by infusing 20 and 350 cSt silicone oil onto S-1, S-2 and S-3 patterns with varying initial top oil layer thickness $(h_{oil})_i$ and initial ridge heights $(h_r)_i$.	3 to 25
Figures S.2, S.4, S.6, S.8, S.10, S.12, S.14, S.16, S.18, S.20, S.22, S.24, S.26, S.28, S.30, S.32, S.34, S.36, S.38, S.40, S.42, S.44, S.46	Plots for the change of liquid-air interfacial area of drops (A_{LV}) during the evaporation of water drops on SLIPS samples which were formed by infusing 20 and 350 cSt silicone oil on S-1, S-2 and S-3 patterns with varying $(h_{oil})_i$ and $(h_r)_i$ values.	3 to 25

Table S.1: The change of the initial ridge height $(h_r)_i$ around the water drop with the increase of initial silicone oil height $(h_{oil})_i$ on the micropatterned samples. (Effective pillar height = 27,5-28,0 μm for S-1 and S-2 and 25,5-26,2 μm for S-3).

**Sample**	**Heights**	**20 cSt silicone oil lubricant** (μm)						**350 cSt silicone oil lubricant** (μm)						
**S-1**	h_{oil}	0,16	2,79	5,42	8,05	10,69	13,32	0,14	2,73	5,31	7,89	10,47	13,06	15,64
	h_r	38	100	120	150	260	342	74	100	120	277	310	370	430
**S-2**	h_{oil}	0,05	2,68	5,32	7,95	13,21	15,84	0,04	2,62	5,20	7,78	10,37	12,95	15,53
	h_r	46	60	73	214	410	430	64	90	119	263	300	323	390
**S-3**	h_{oil}	0,01	2,63	5,26	7,89	13,15	15,79	0,16	2,74	5,33	7,91	10,49	13,07	15,66
	h_r	67	120	184	280	439	530	121	190	291	324	400	500	580

Table S.2: The change of initial ridge height $(h_r)_i$ around the water drop after half life-time during evaporation on the micropatterned SLIPS samples infused by 20 cSt silicone oil.

**Sample**	h_{oil} (μm)	$(h_r)_{initial}$ (μm)	$(h_r)_{half\ life\text{-}time}$ (μm)	Δh_r (μm)
**S-1**	0,16	33	55	22
**S-2**	5,32	73	83	10
**S-3**	0,01	66	74	8

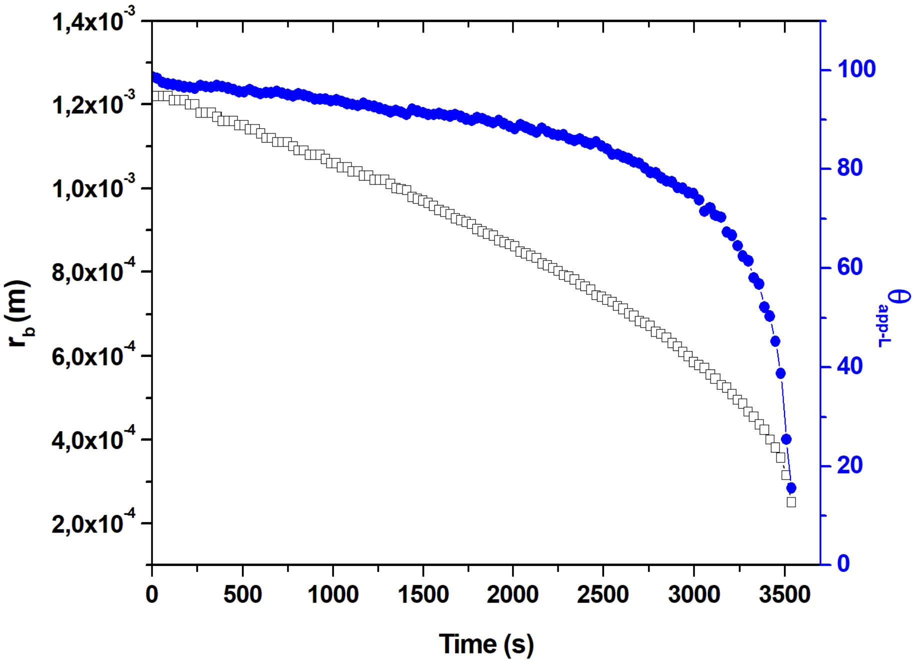

Figure S.1. The change of contact radius (r_b) and apparent contact angle at the liquid three-phase contact line at the lubricant-water-air phases (θ_{app-L}) during the evaporation of a water drop placed on a SLIPS sample formed by infusing 20 cSt silicone oil on the S-1 sample with a top oil layer thickness $(h_{oil})_i$ = 5,47 μm and initial ridge height $(h_r)_i$ = 119 μm.

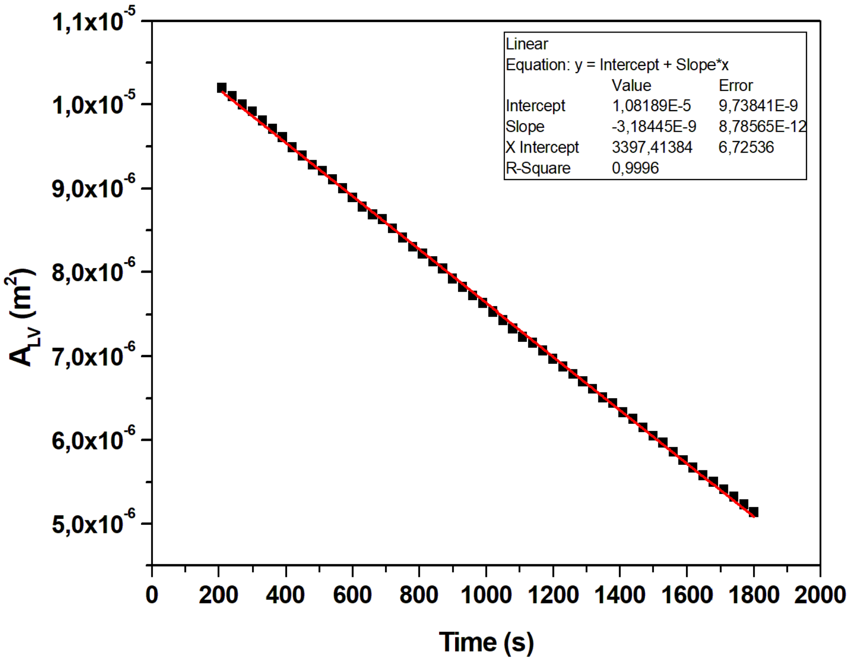

Figure S.2. The change of liquid-air interfacial area of water drop (A_{LV}) during the evaporation of a water drop on a SLIPS sample formed by infusing 20 cSt silicone oil on the S-1 sample with a top oil layer thickness $(h_{oil})_i$ = 5,47 μm and initial ridge height $(h_r)_i$ = 119 μm.

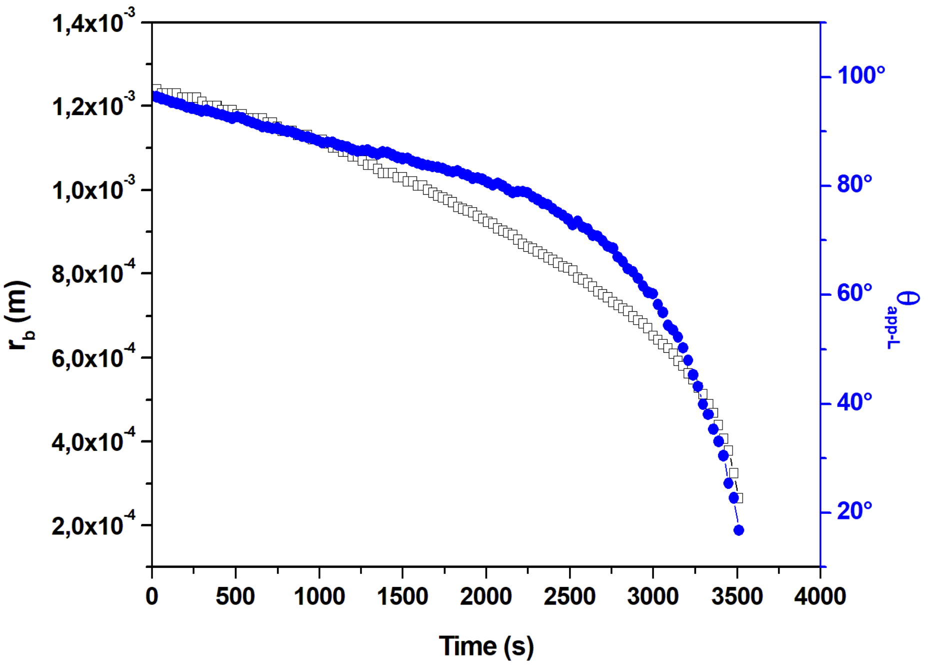

Figure S.3. The change of contact radius (r_b) and apparent contact angle at the liquid three-phase contact line at the lubricant-water-air phases ($\theta_{app\text{-}L}$) during the evaporation of a water drop placed on a SLIPS sample formed by infusing 20 cSt silicone oil on the S-1 sample with a top oil layer thickness $(h_{oil})_i$ = 8,06 μm and initial ridge height $(h_r)_i$ = 146 μm.

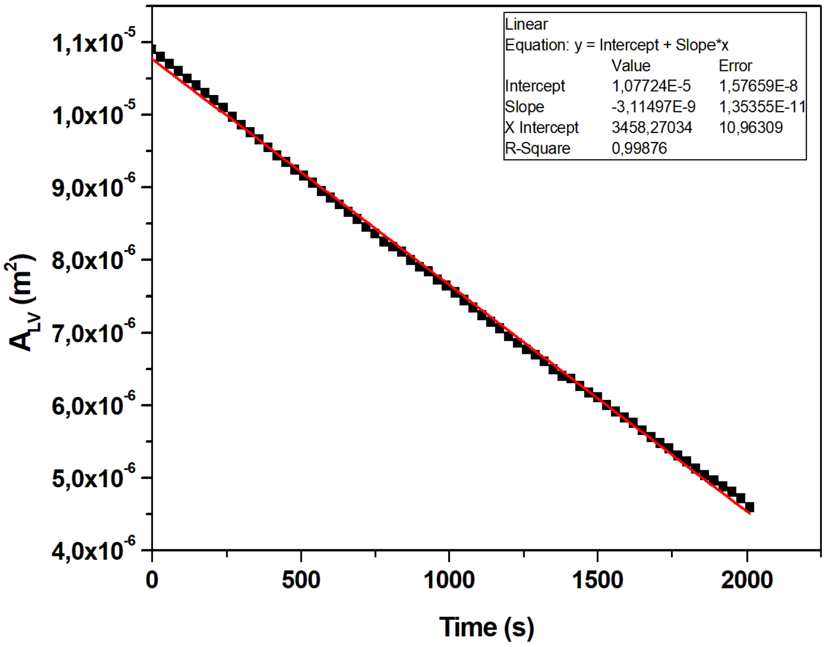

Figure S.4. The change of liquid-air interfacial area of water drop (A_{LV}) during the evaporation of a water drop on a SLIPS sample formed by infusing 20 cSt silicone oil on the S-1 sample with a top oil layer thickness $(h_{oil})_i$ = 8,06 μm and initial ridge height $(h_r)_i$ = 146 μm.

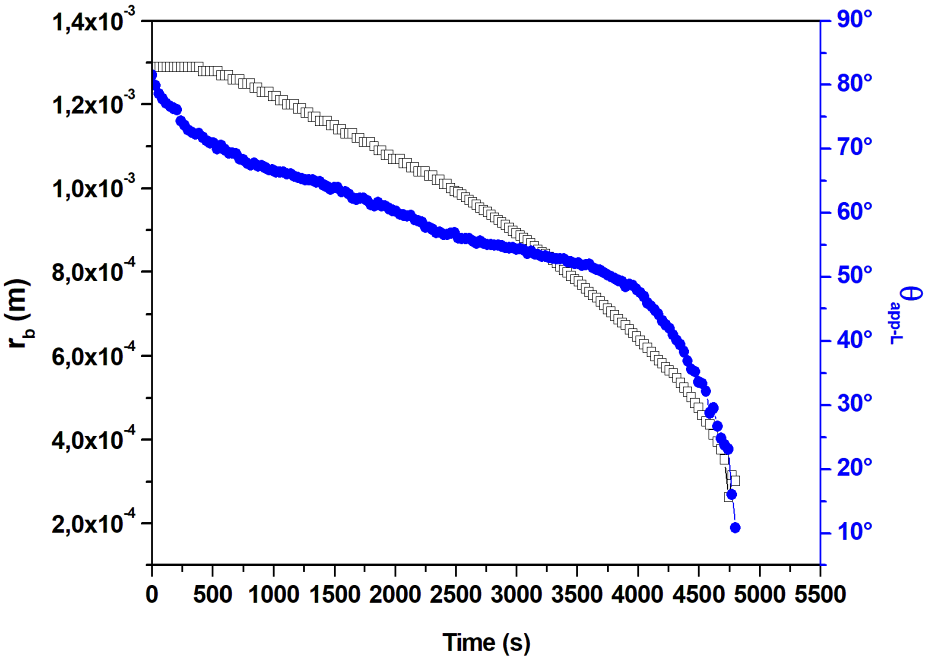

Figure S.5. The change of contact radius (r_b) and apparent contact angle at the liquid three-phase contact line at the lubricant-water-air phases ($\theta_{app\text{-}L}$) during the evaporation of a water drop placed on a SLIPS sample formed by infusing 20 cSt silicone oil on the S-1 sample with a top oil layer thickness $(h_{oil})_i$ = 13,30 μm and initial ridge height $(h_r)_i$ = 333 μm.

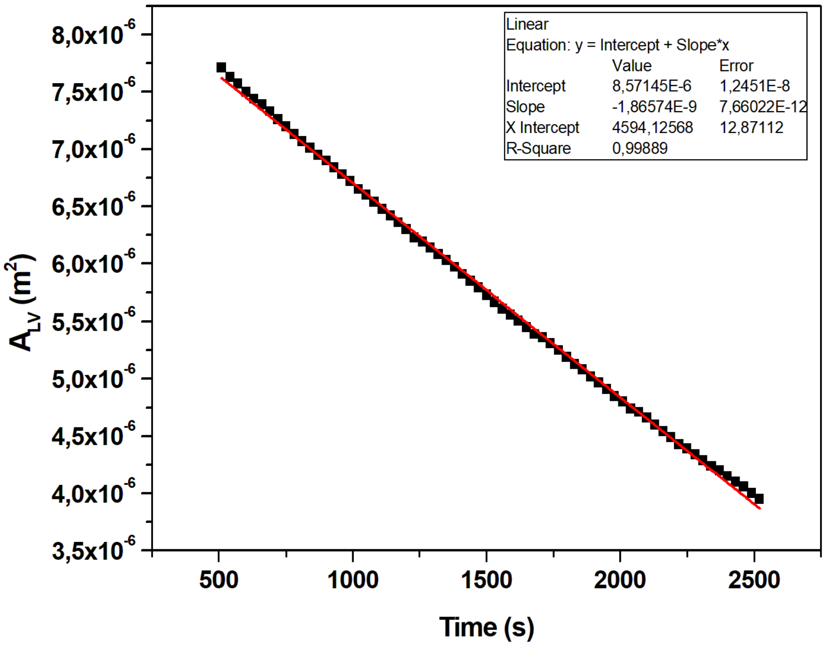

Figure S.6. The change of liquid-air interfacial area of water drop (A_{LV}) during the evaporation of a water drop on a SLIPS sample formed by infusing 20 cSt silicone oil on the S-1 sample with a top oil layer thickness $(h_{oil})_i$ = 13,30 μm and initial ridge height $(h_r)_i$ = 333 μm.

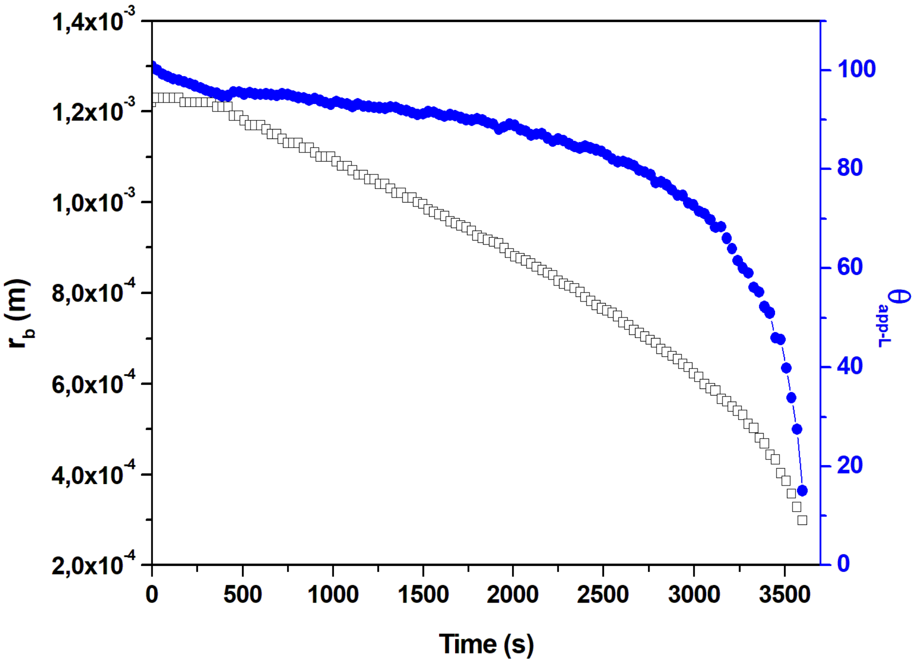

Figure S.7. The change of contact radius (r_b) and apparent contact angle at the liquid three-phase contact line at the lubricant-water-air phases (θ_{app-L}) during the evaporation of a water drop placed on a SLIPS sample formed by infusing 350 cSt silicone oil on the S-1 sample with a top oil layer thickness $(h_{oil})_i$ = 0,14 μm and initial ridge height $(h_r)_i$ = 74 μm.

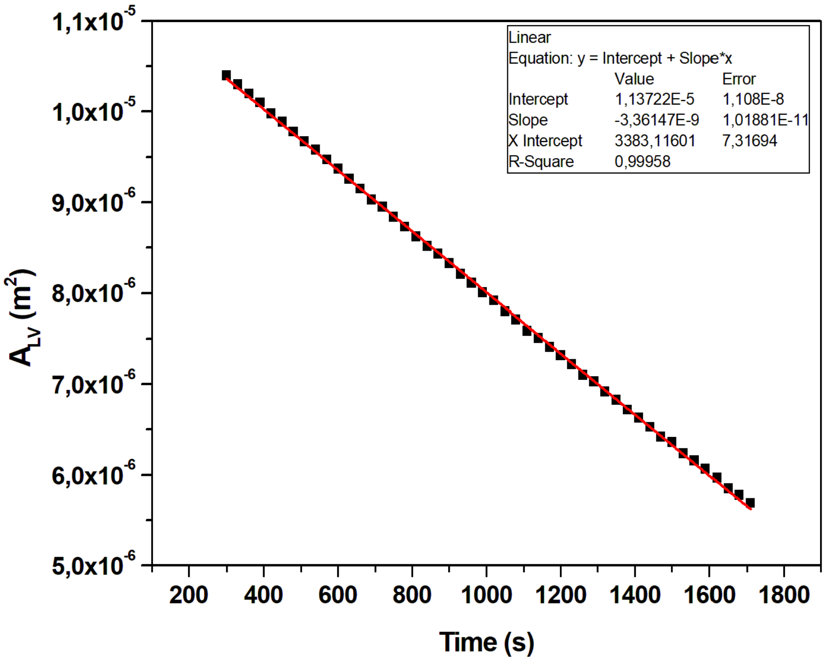

Figure S.8. The change of liquid-air interfacial area of water drop (A_{LV}) during the evaporation of a water drop on a SLIPS sample formed by infusing 350 cSt silicone oil on the S-1 sample with a top oil layer thickness $(h_{oil})_i$ = 0,14 μm) and initial ridge height $(h_r)_i$ = 74 μm.

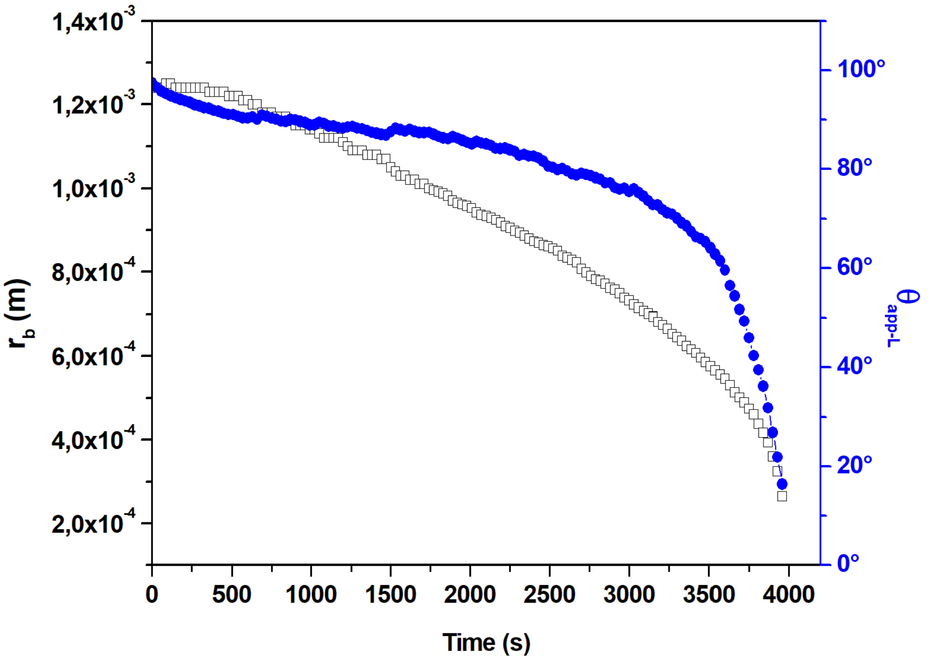

Figure S.9. The change of contact radius (r_b) and apparent contact angle at the liquid three-phase contact line at the lubricant-water-air phases ($\theta_{app\text{-}L}$) during the evaporation of a water drop placed on a SLIPS sample formed by infusing 350 cSt silicone oil on the S-1 sample with a top oil layer thickness $(h_{oil})_i$ = 5,35 μm and initial ridge height $(h_r)_i$ = 129 μm.

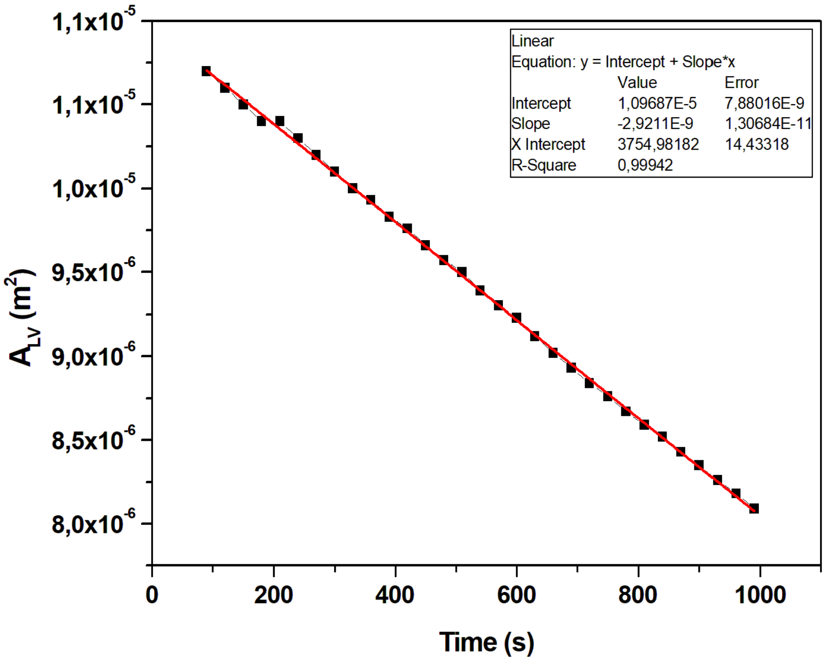

Figure S.10. The change of liquid-air interfacial area of water drop (A_{LV}) during the evaporation of a water drop on a SLIPS sample formed by infusing 350 cSt silicone oil on the S-1 sample with a top oil layer thickness $(h_{oil})_i$ = 5,35 μm and initial ridge height $(h_r)_i$ = 129 μm.

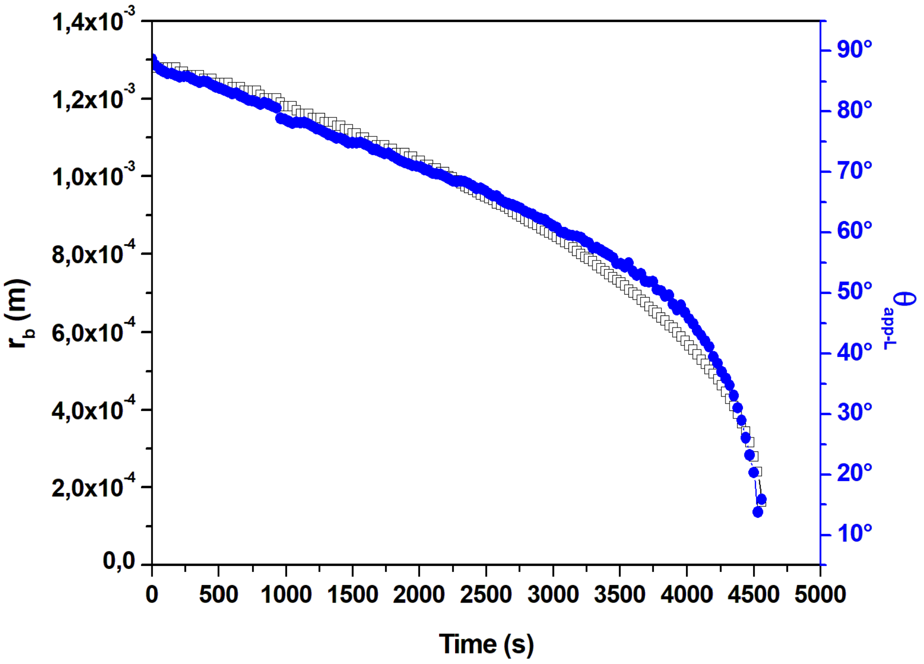

Figure S.11. The change of contact radius (r_b) and apparent contact angle at the liquid three-phase contact line at the lubricant-water-air phases ($\theta_{app\text{-}L}$) during the evaporation of a water drop placed on a SLIPS sample formed by infusing 350 cSt silicone oil on the S-1 sample with a top oil layer thickness $(h_{oil})_i$ = 7,89 μm and initial ridge height $(h_r)_i$ = 277 μm.

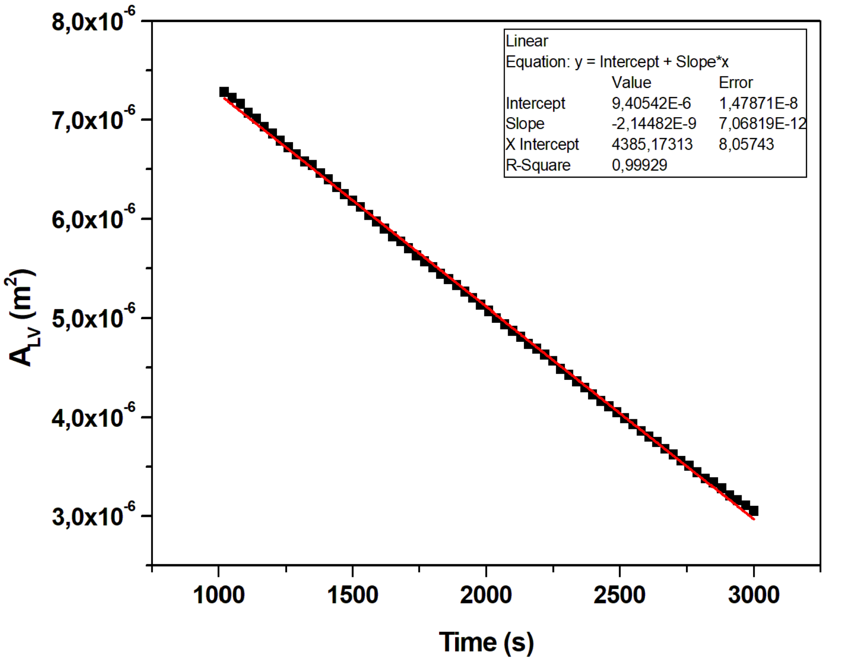

Figure S.12. The change of liquid-air interfacial area of water drop (A_{LV}) during the evaporation of a water drop on a SLIPS sample formed by infusing 350 cSt silicone oil on the S-1 sample with a top oil layer thickness $(h_{oil})_i$ = 7,89 μm and initial ridge height $(h_r)_i$ = 277 μm.

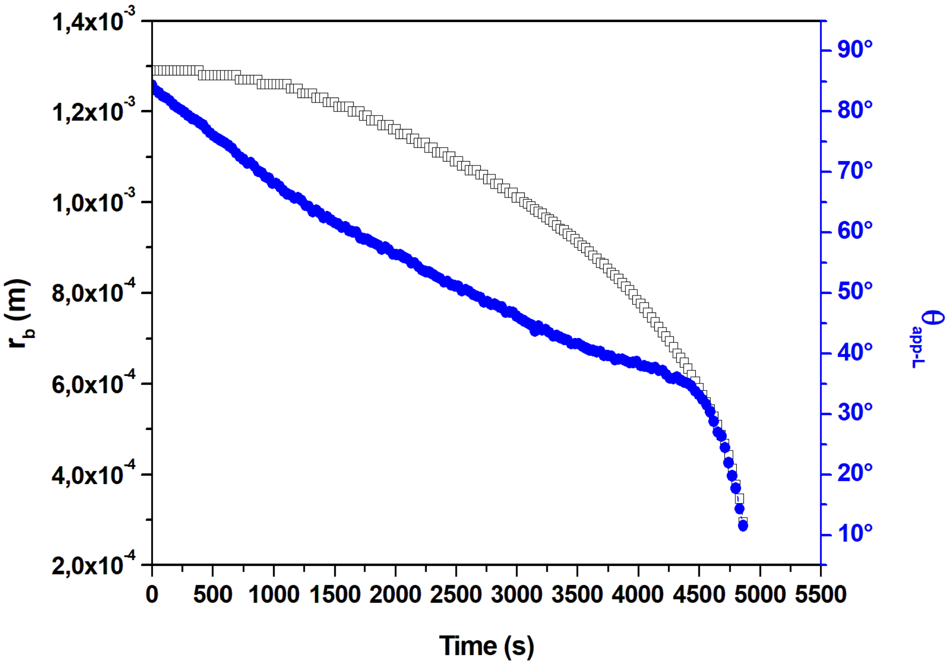

Figure S.13. The change of contact radius (r_b) and apparent contact angle at the liquid three-phase contact line at the lubricant-water-air phases ($\theta_{app\text{-}L}$) during the evaporation of a water drop placed on a SLIPS sample formed by infusing 350 cSt silicone oil on the S-1 sample with a top oil layer thickness $(h_{oil})_i$ = 13,06 μm and initial ridge height $(h_r)_i$ = 310 μm.

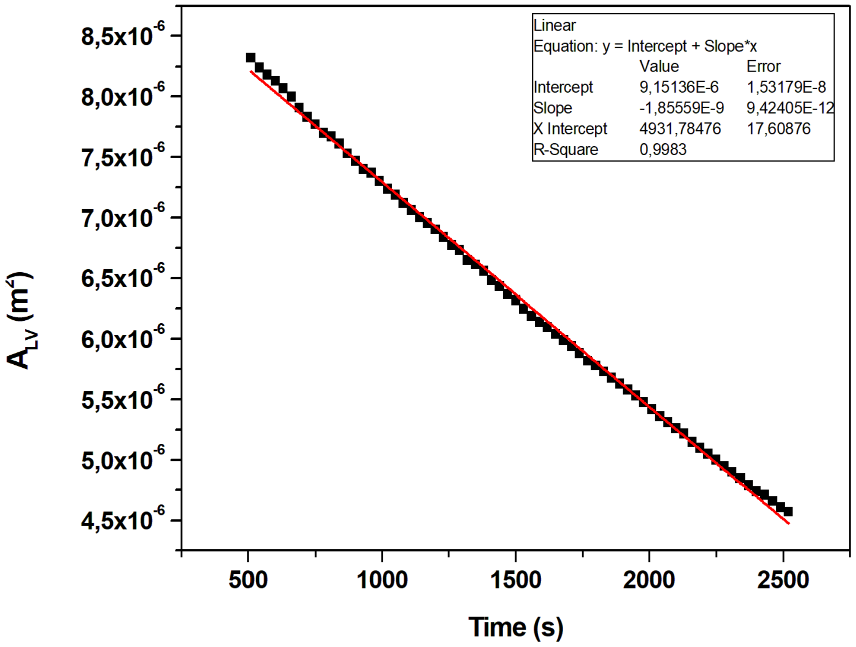

Figure S.14. The change of liquid-air interfacial area of water drop (A_{LV}) during the evaporation of a water drop on a SLIPS sample formed by infusing 350 cSt silicone oil on the S-1 sample with a top oil layer thickness $(h_{oil})_i$ = 13,06 μm and initial ridge height $(h_r)_i$ = 310 μm.

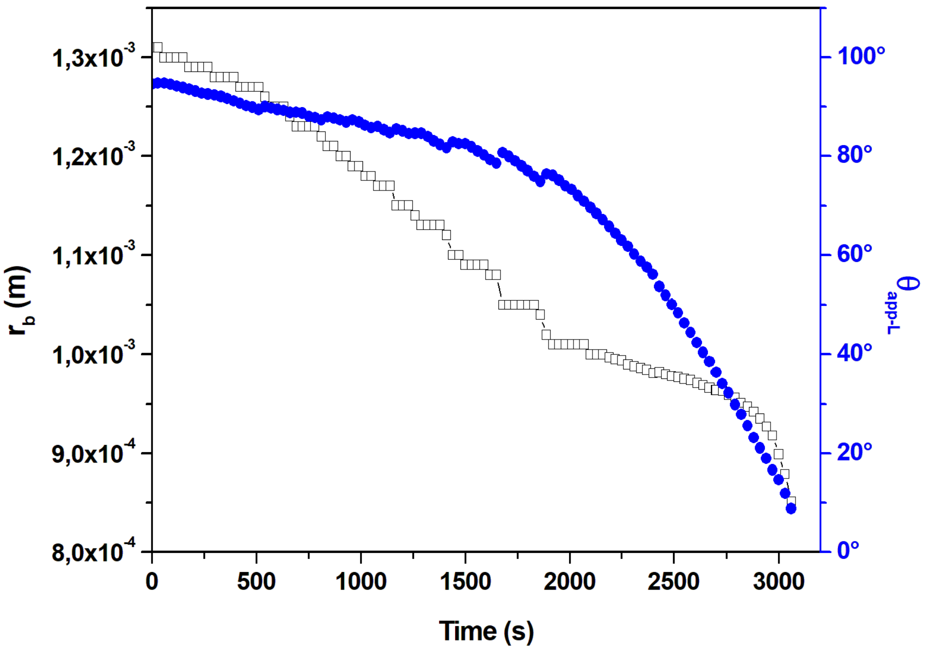

Figure S.15. The change of contact radius (r_b) and apparent contact angle at the liquid three-phase contact line at the lubricant-water-air phases (θ_{app-L}) during the evaporation of a water drop placed on a SLIPS sample formed by infusing 20 cSt silicone oil on the S-2 sample with a top oil layer thickness $(h_{oil})_i$ = 0,05 μm and initial ridge height $(h_r)_i$ = 46 μm.

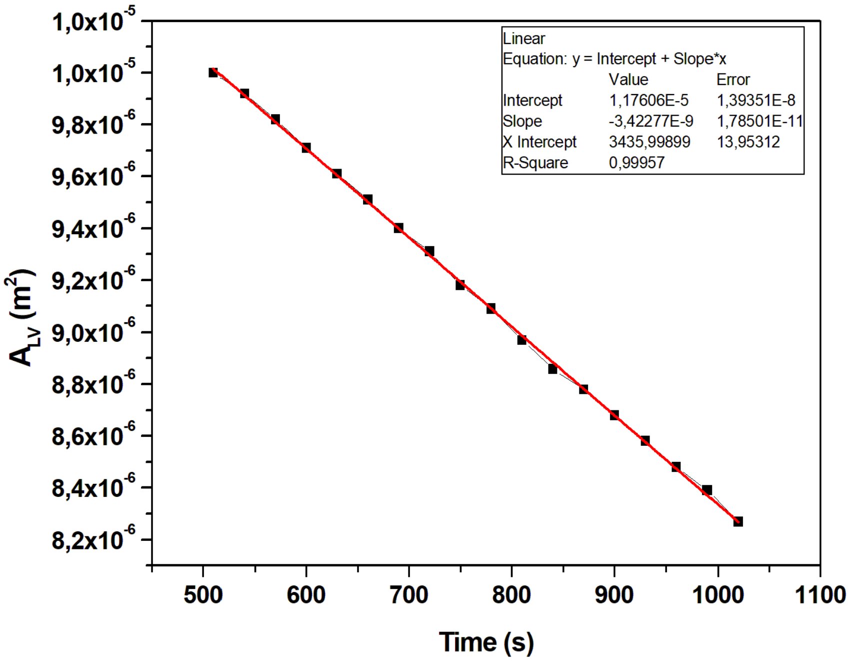

Figure S.16. The change of liquid-air interfacial area of water drop (A_{LV}) during the evaporation of a water drop on a SLIPS sample formed by infusing 20 cSt silicone oil on the S-2 sample with a top oil layer thickness $(h_{oil})_i$ = 0,05 μm and initial ridge height $(h_r)_i$ = 46 μm.

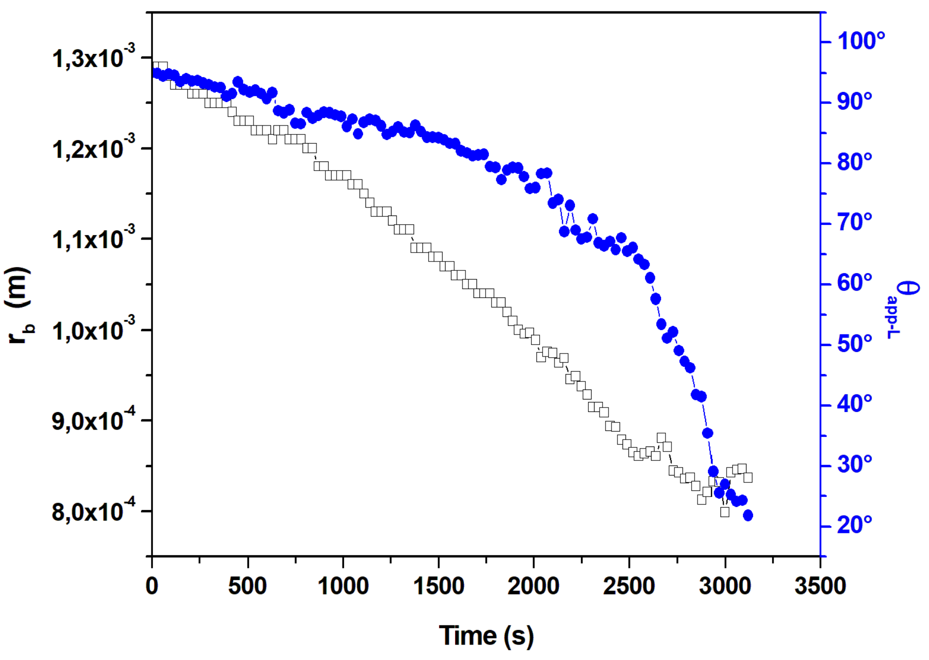

Figure S.17. The change of contact radius (r_b) and apparent contact angle at the liquid three-phase contact line at the lubricant-water-air phases ($\theta_{app\text{-}L}$) during the evaporation of a water drop placed on a SLIPS sample formed by infusing 20 cSt silicone oil on the S-2 sample with a top oil layer thickness $(h_{oil})_i$ = 5,32 μm and initial ridge height $(h_r)_i$ = 73 μm.

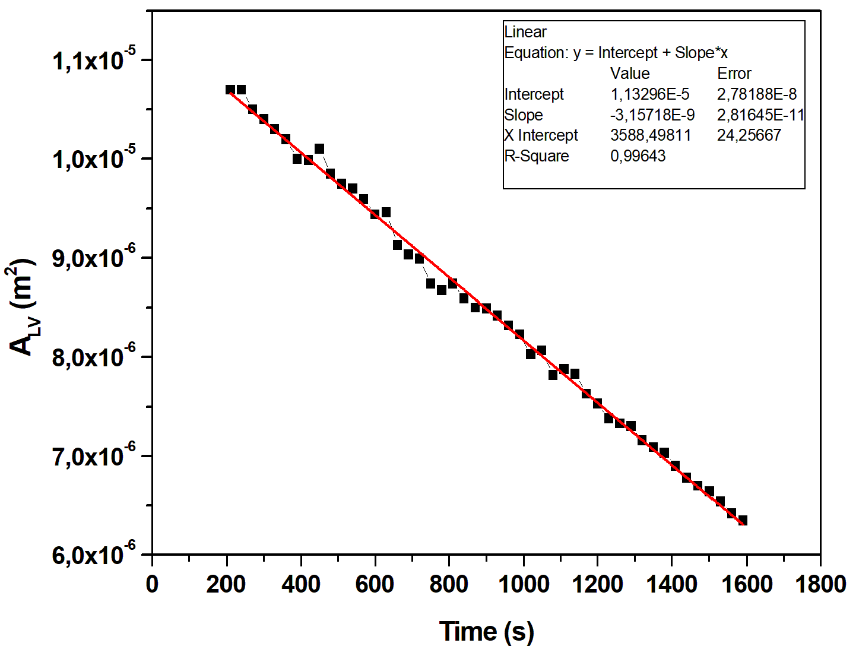

Figure S.18. The change of liquid-air interfacial area of water drop (A_{LV}) during the evaporation of a water drop on a SLIPS sample formed by infusing 20 cSt silicone oil on the S-2 sample with a top oil layer thickness $(h_{oil})_i$ = 5,32 μm and initial ridge height $(h_r)_i$ = 73 μm.

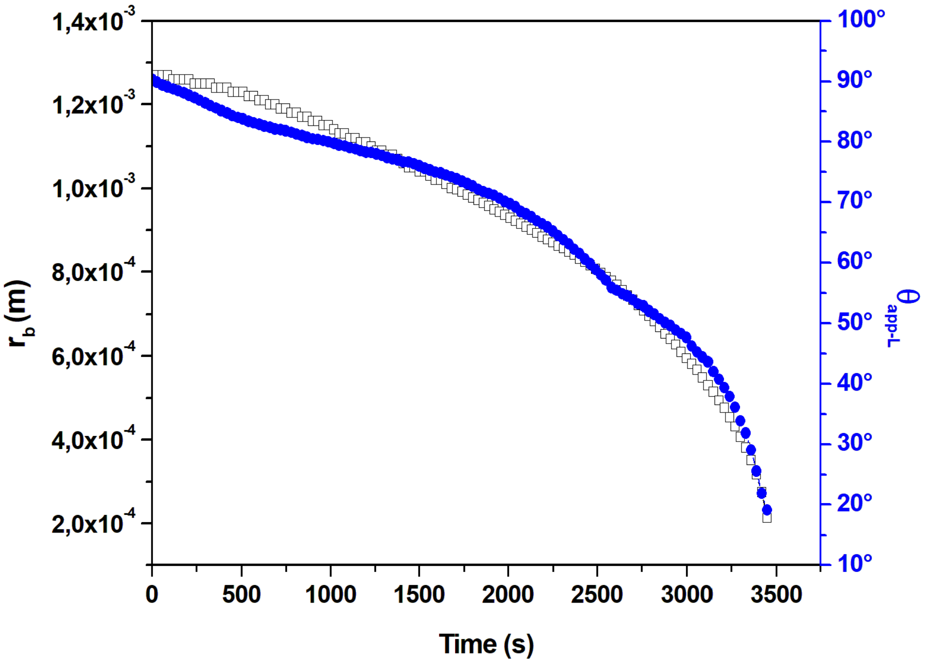

Figure S.19. The change of contact radius (r_b) and apparent contact angle at the liquid three-phase contact line at the lubricant-water-air phases (θ_{app-L}) during the evaporation of a water drop placed on a SLIPS sample formed by infusing 20 cSt silicone oil on the S-2 sample with a top oil layer thickness $(h_{oil})_i$ = 7,90 μm and initial ridge height $(h_r)_i$ = 193 μm.

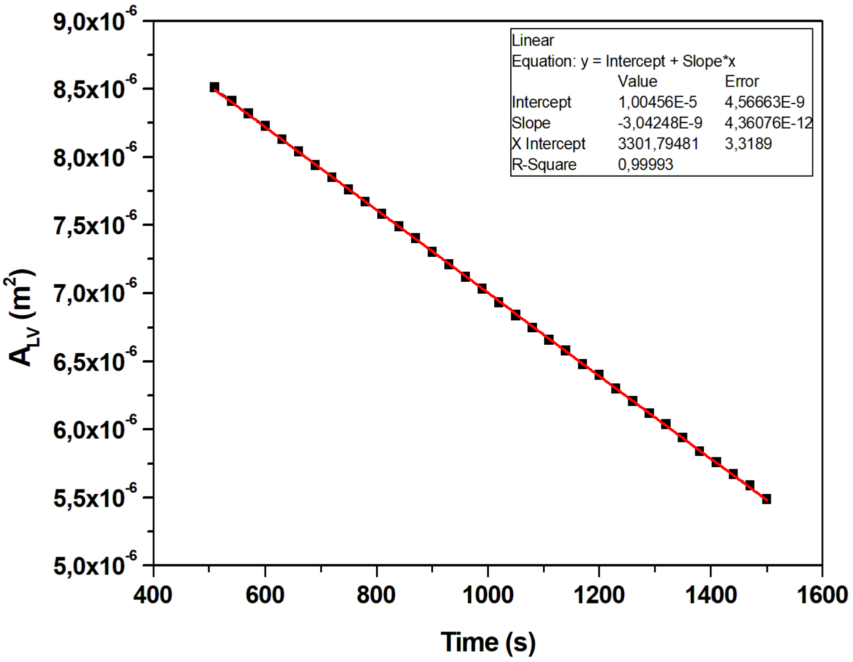

Figure S.20. The change of liquid-air interfacial area of water drop (A_{LV}) during the evaporation of a water drop on a SLIPS sample formed by infusing 20 cSt silicone oil on the S-2 sample with a top oil layer thickness $(h_{oil})_i$ = 7,90 μm and initial ridge height $(h_r)_i$ = 193 μm.

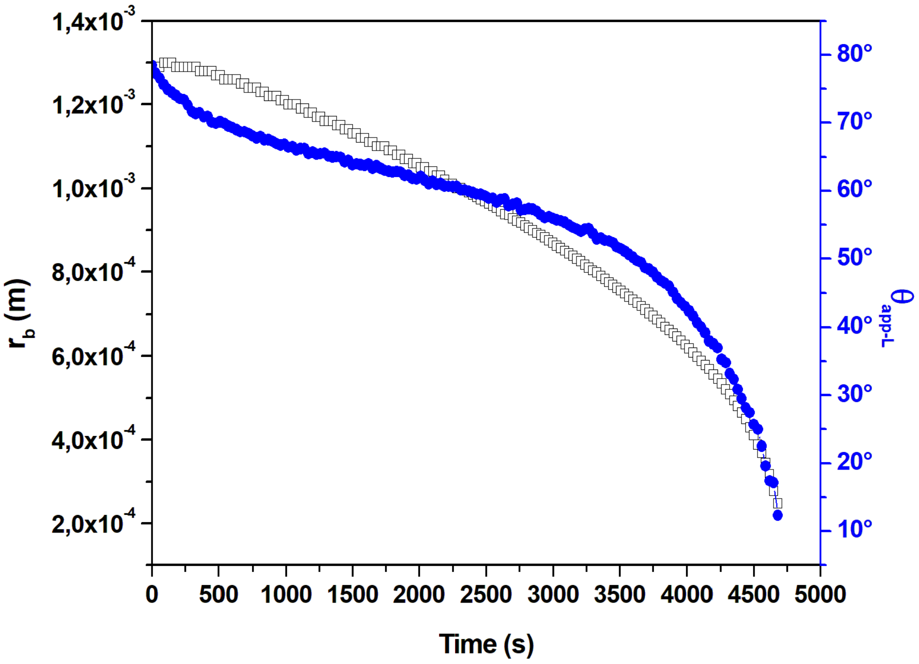

Figure S.21. The change of contact radius (r_b) and apparent contact angle at the liquid three-phase contact line at the lubricant-water-air phases ($\theta_{app\text{-}L}$) during the evaporation of a water drop placed on a SLIPS sample formed by infusing 20 cSt silicone oil on the S-2 sample with a top oil layer thickness $(h_{oil})_i$ = 13,21 μm and initial ridge height $(h_r)_i$ = 448 μm.

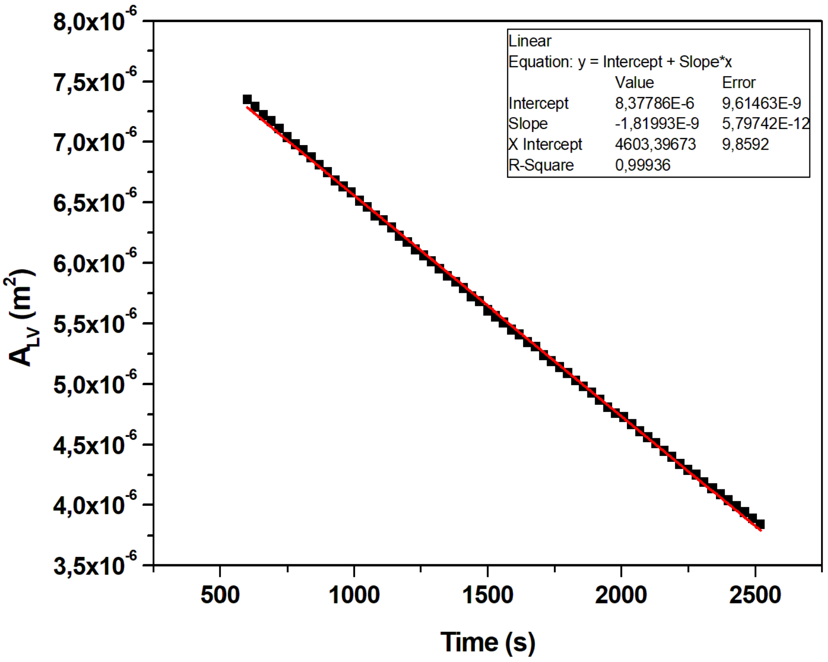

Figure S.22. The change of liquid-air interfacial area of water drop (A_{LV}) during the evaporation of a water drop on a SLIPS sample formed by infusing 20 cSt silicone oil on the S-2 sample with a top oil layer thickness $(h_{oil})_i$ = 13,21 μm and initial ridge height $(h_r)_i$ = 448 μm.

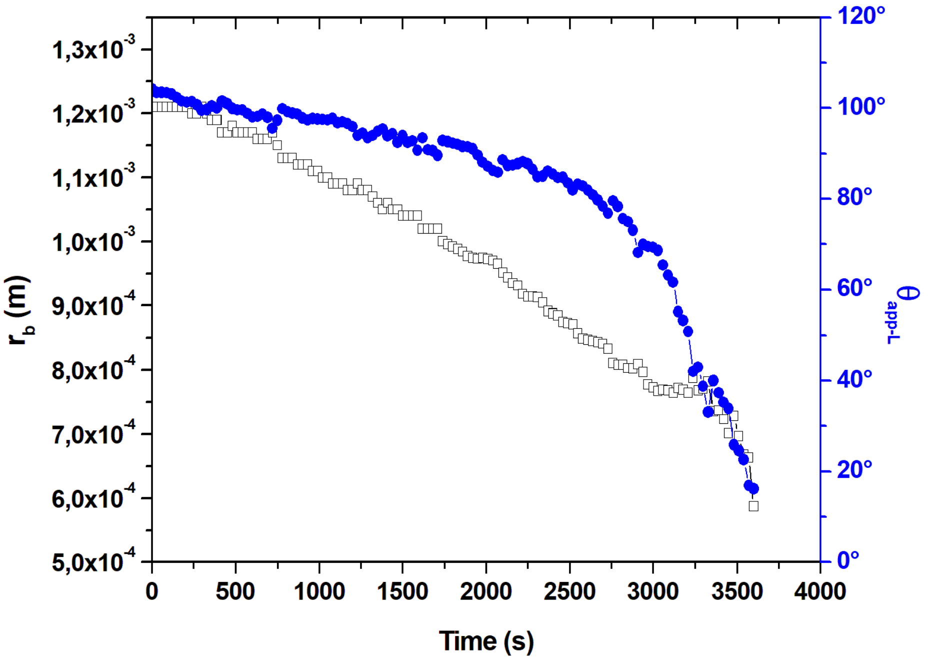

Figure S.23. The change of contact radius (r_b) and apparent contact angle at the liquid three-phase contact line at the lubricant-water-air phases ($\theta_{app\text{-}L}$) during the evaporation of a water drop placed on a SLIPS sample formed by infusing 350 cSt silicone oil on the S-2 sample with a top oil layer thickness $(h_{oil})_i$ = 0,03 μm and initial ridge height $(h_r)_i$ = 37 μm.

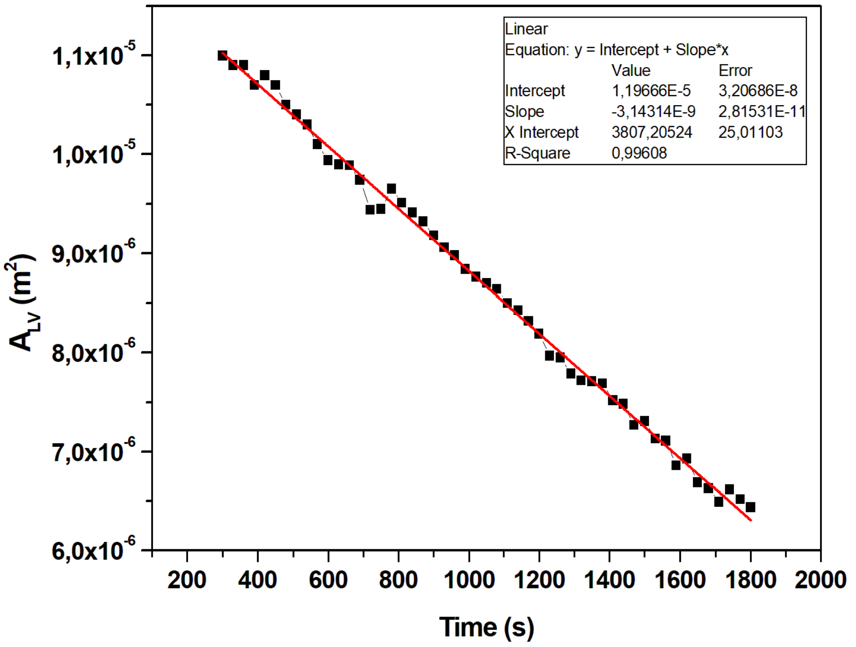

Figure S.24. The change of liquid-air interfacial area of water drop (A_{LV}) during the evaporation of a water drop on a SLIPS sample formed by infusing 350 cSt silicone oil on the S-2 sample with a top oil layer thickness $(h_{oil})_i$ = 0,03 μm and initial ridge height $(h_r)_i$ = 37 μm.

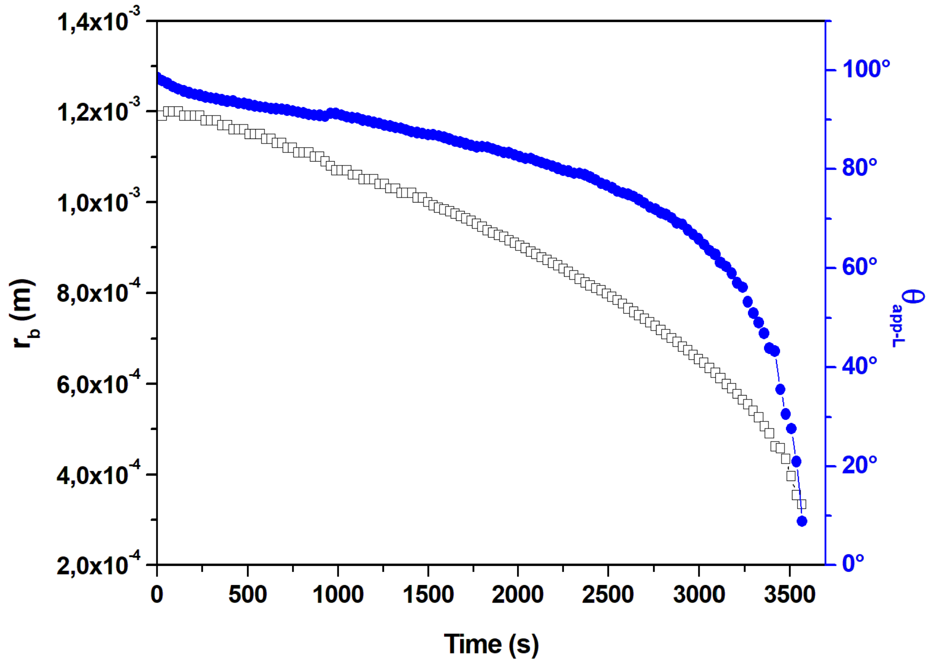

Figure S.25. The change of contact radius (r_b) and apparent contact angle at the liquid three-phase contact line at the lubricant-water-air phases ($\theta_{app\text{-}L}$) during the evaporation of a water drop placed on a SLIPS sample formed by infusing 350 cSt silicone oil on the S-2 sample with a top oil layer thickness $(h_{oil})_i$ = 3,20 μm and initial ridge height $(h_r)_i$ = 67 μm.

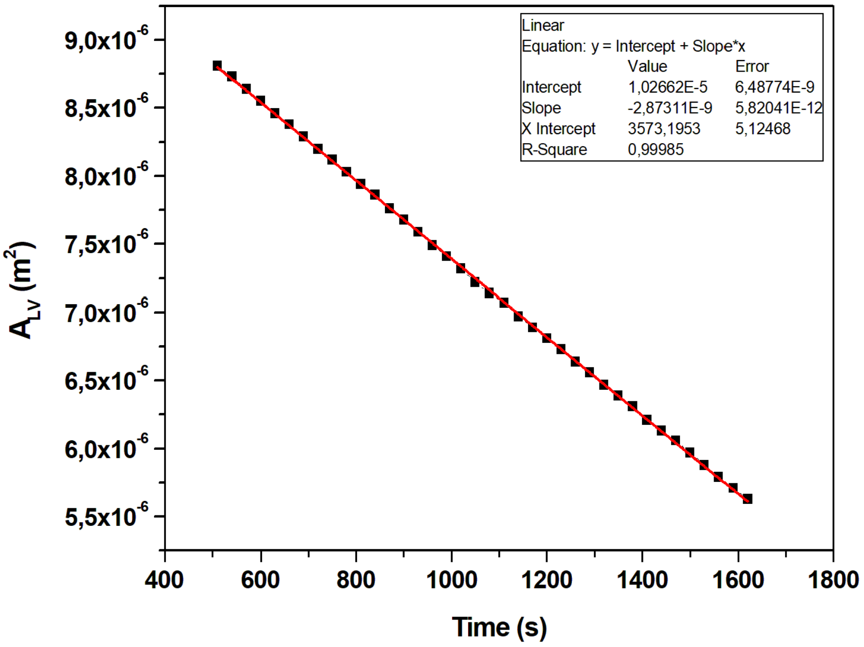

Figure S.26. The change of liquid-air interfacial area of water drop (A_{LV}) during the evaporation of a water drop on a SLIPS sample formed by infusing 350 cSt silicone oil on the S-2 sample with a top oil layer thickness $(h_{oil})_i$ = 3,20 μm and initial ridge height $(h_r)_i$ = 67 μm.

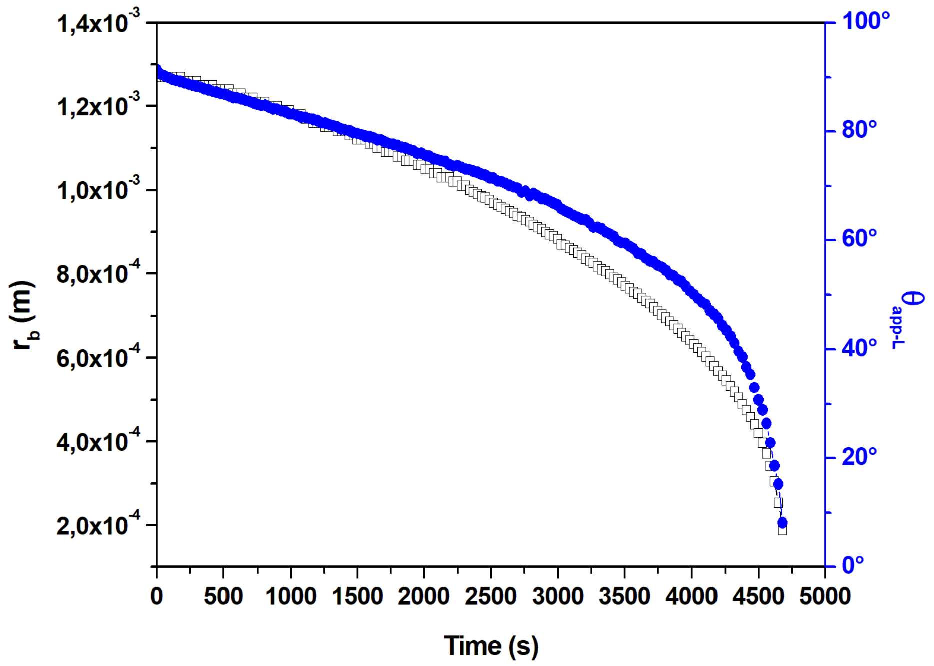

Figure S.27. The change of contact radius (r_b) and apparent contact angle at the liquid three-phase contact line at the lubricant-water-air phases (θ_{app-L}) during the evaporation of a water drop placed on a SLIPS sample formed by infusing 350 cSt silicone oil on the S-2 sample with a top oil layer thickness $(h_{oil})_i$ = 7,75 μm and initial ridge height $(h_r)_i$ = 233 μm.

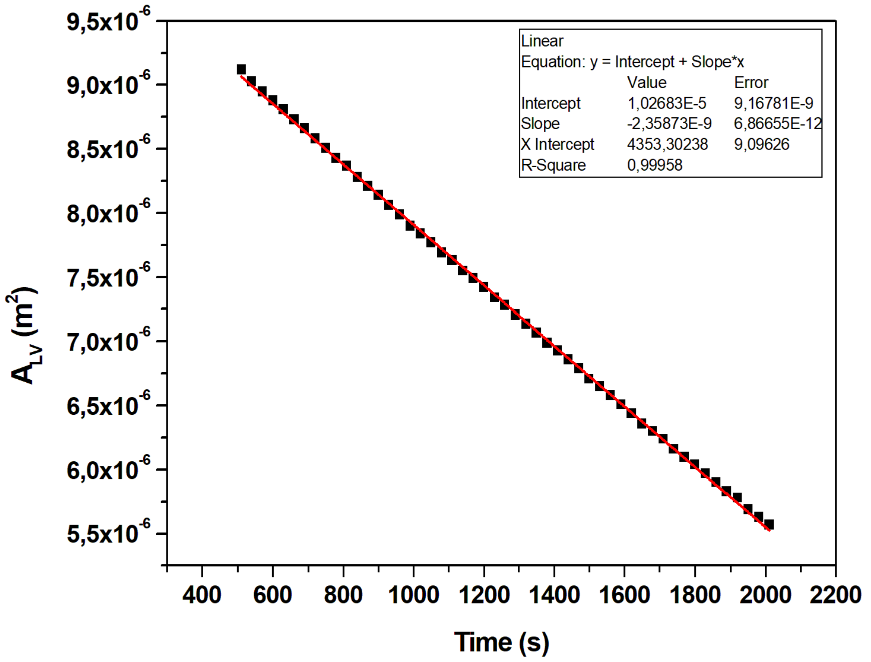

Figure S.28. The change of liquid-air interfacial area of water drop (A_{LV}) during the evaporation of a water drop on a SLIPS sample formed by infusing 350 cSt silicone oil on the S-2 sample with a top oil layer thickness $(h_{oil})_i$ = 7,75 μm and initial ridge height $(h_r)_i$ = 233 μm.

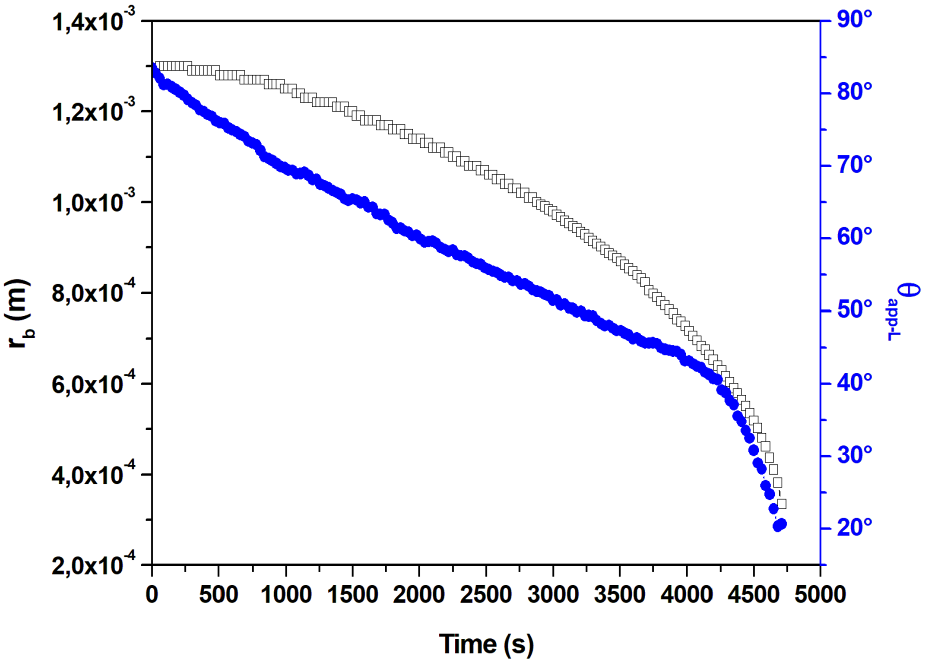

Figure S.29. The change of contact radius (r_b) and apparent contact angle at the liquid three-phase contact line at the lubricant-water-air phases ($\theta_{app\text{-}L}$) during the evaporation of a water drop placed on a SLIPS sample formed by infusing 350 cSt silicone oil on the S-2 sample with a top oil layer thickness $(h_{oil})_i$ = 12,96 μm and initial ridge height $(h_r)_i$ = 328 μm.

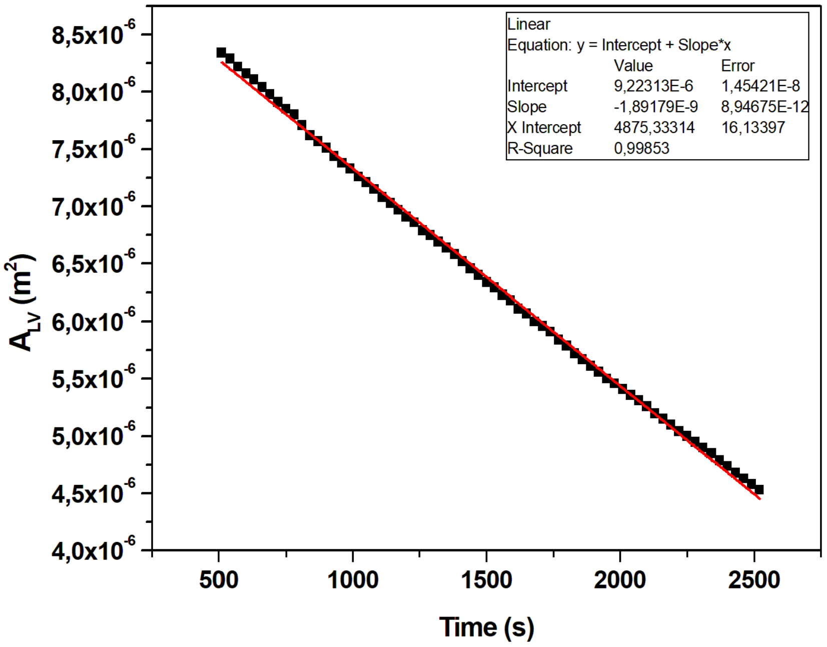

Figure S.30. The change of liquid-air interfacial area of water drop (A_{LV}) during the evaporation of a water drop on a SLIPS sample formed by infusing 350 cSt silicone oil on the S-2 sample with a top oil layer thickness $(h_{oil})_i$ = 12,96 μm and initial ridge height $(h_r)_i$ = 328 μm.

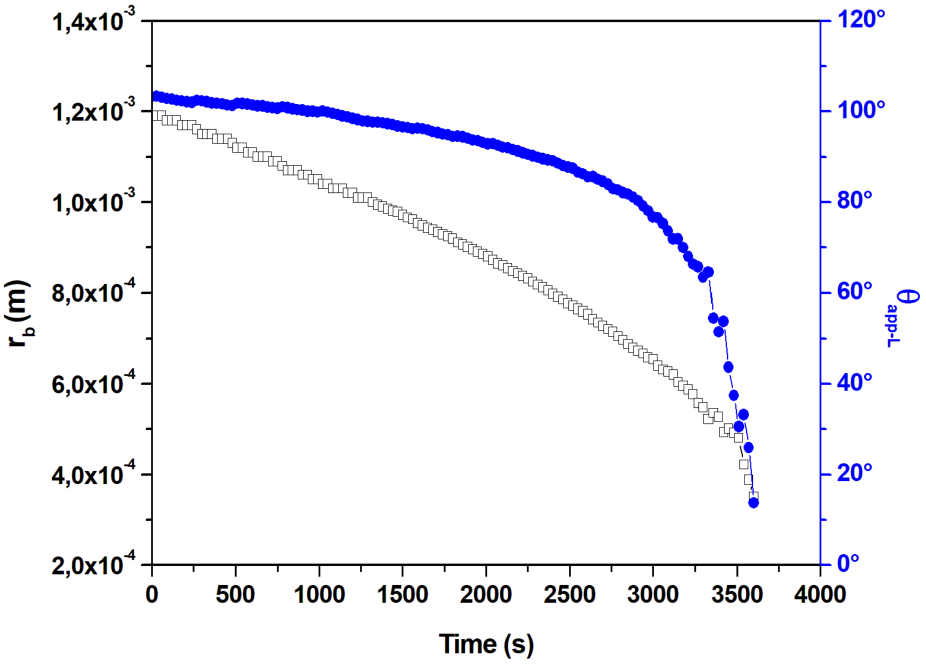

Figure S.31. The change of contact radius (r_b) and apparent contact angle at the liquid three-phase contact line at the lubricant-water-air phases ($\theta_{app\text{-}L}$) during the evaporation of a water drop placed on a SLIPS sample formed by infusing 20 cSt silicone oil on the S-3 sample with a top oil layer thickness $(h_{oil})_i$ = 0,01 μm and initial ridge height $(h_r)_i$ = 66 μm.

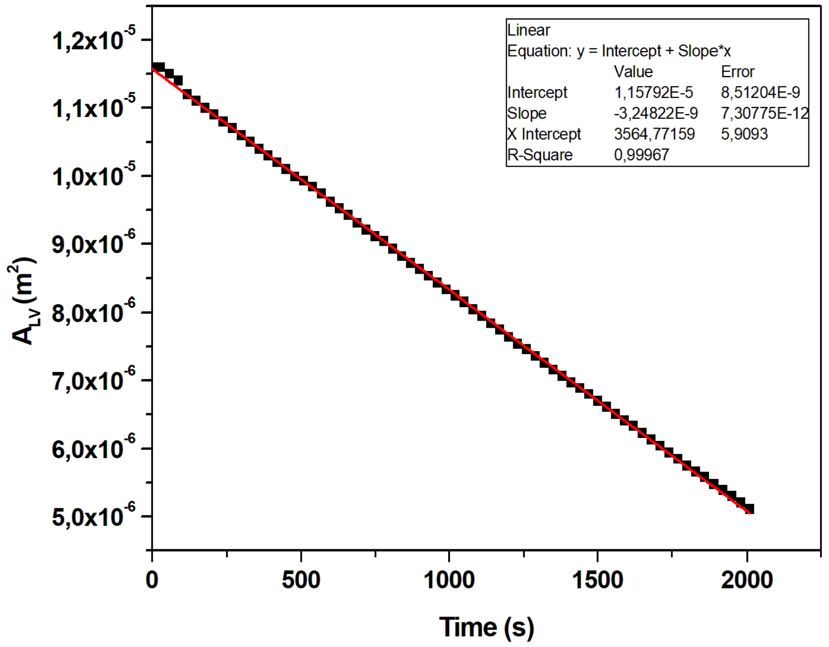

Figure S.32. The change of liquid-air interfacial area of water drop (A_{LV}) during the evaporation of a water drop on a SLIPS sample formed by infusing 20 cSt silicone oil on the S-3 sample with a top oil layer thickness $(h_{oil})_i$ = 0,01 μm and initial ridge height $(h_r)_i$ = 66 μm.

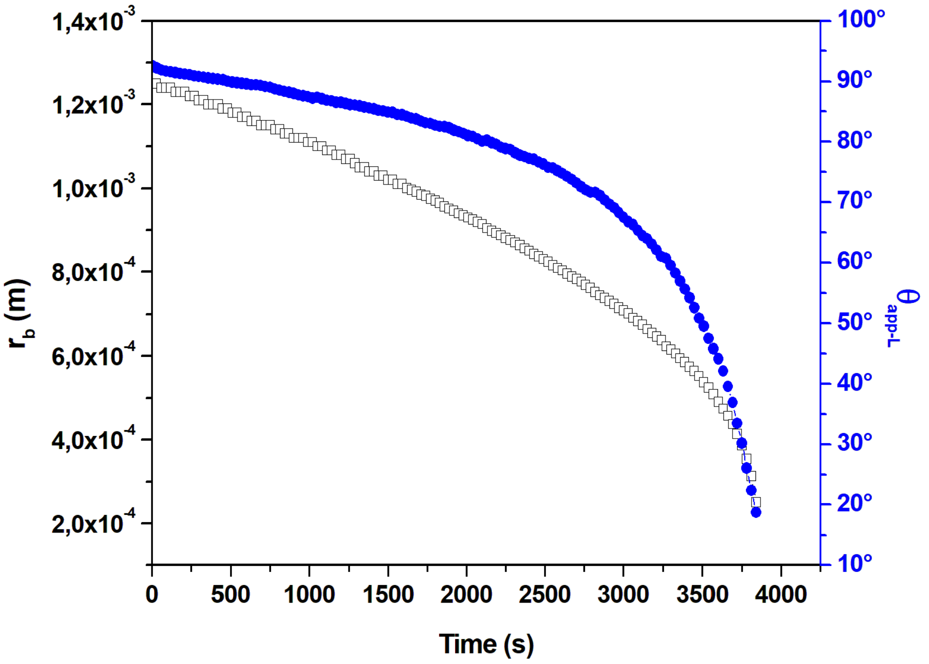

Figure S.33. The change of contact radius (r_b) and apparent contact angle at the liquid three-phase contact line at the lubricant-water-air phases ($\theta_{app\text{-}L}$) during the evaporation of a water drop placed on a SLIPS sample formed by infusing 20 cSt silicone oil on the S-3 sample with a top oil layer thickness $(h_{oil})_i$ = 5,26 μm and initial ridge height $(h_r)_i$ = 184 μm.

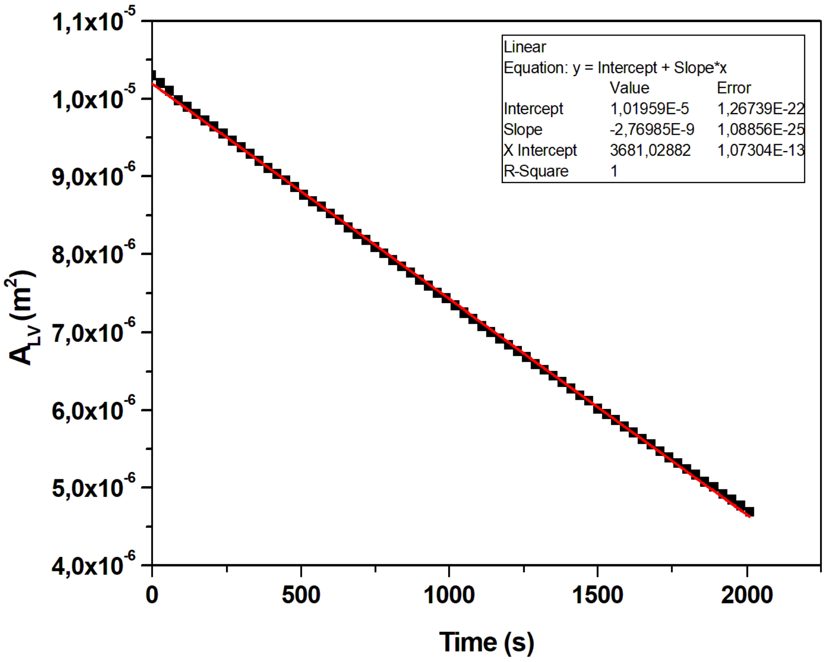

Figure S.34. The change of liquid-air interfacial area of water drop (A_{LV}) during the evaporation of a water drop on a SLIPS sample formed by infusing 20 cSt silicone oil on the S-3 sample with a top oil layer thickness $(h_{oil})_i$ = 5,26 μm and initial ridge height $(h_r)_i$ = 184 μm.

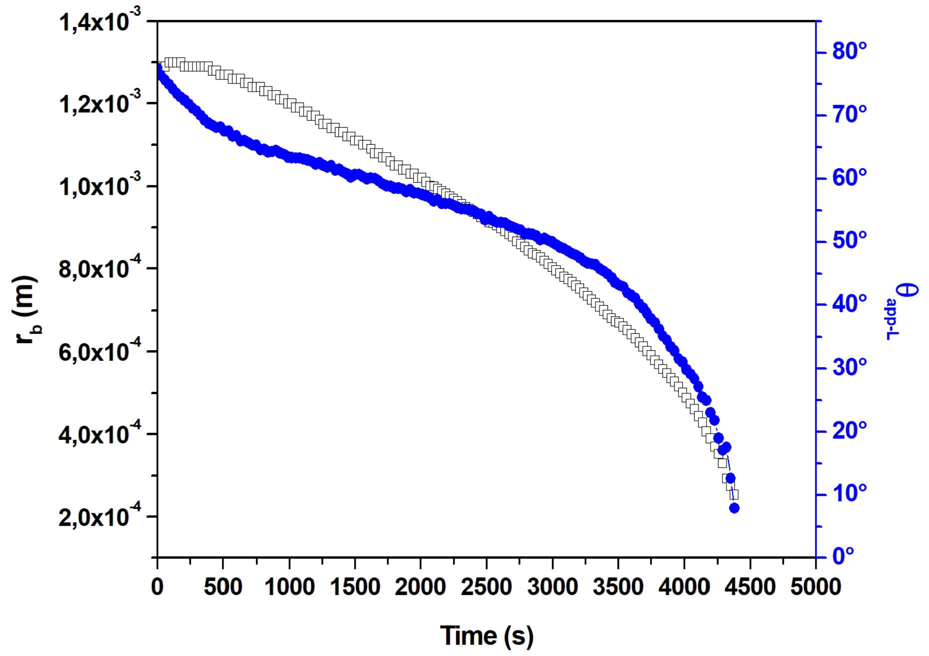

Figure S.35. The change of contact radius (r_b) and apparent contact angle at the liquid three-phase contact line at the lubricant-water-air phases (θ_{app-L}) during the evaporation of a water drop placed on a SLIPS sample formed by infusing 20 cSt silicone oil on the S-3 sample with a top oil layer thickness $(h_{oil})_i$ = 7,87 µm and initial ridge height $(h_r)_i$ = 280 µm.

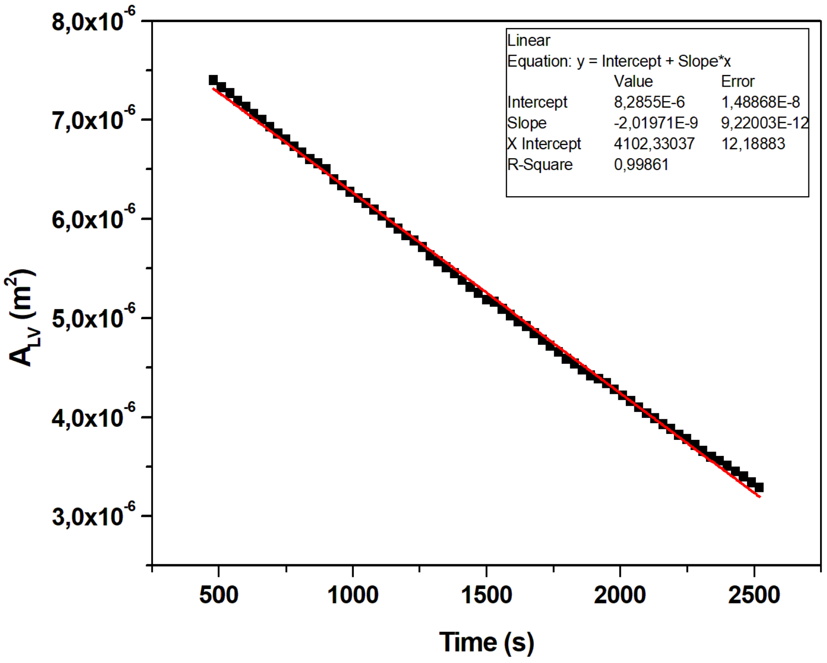

Figure S.36. The change of liquid-air interfacial area of water drop (A_{LV}) during the evaporation of a water drop on a SLIPS sample formed by infusing 20 cSt silicone oil on the S-3 sample with a top oil layer thickness $(h_{oil})_i$ = 7,87 µm and initial ridge height $(h_r)_i$ = 280 µm.

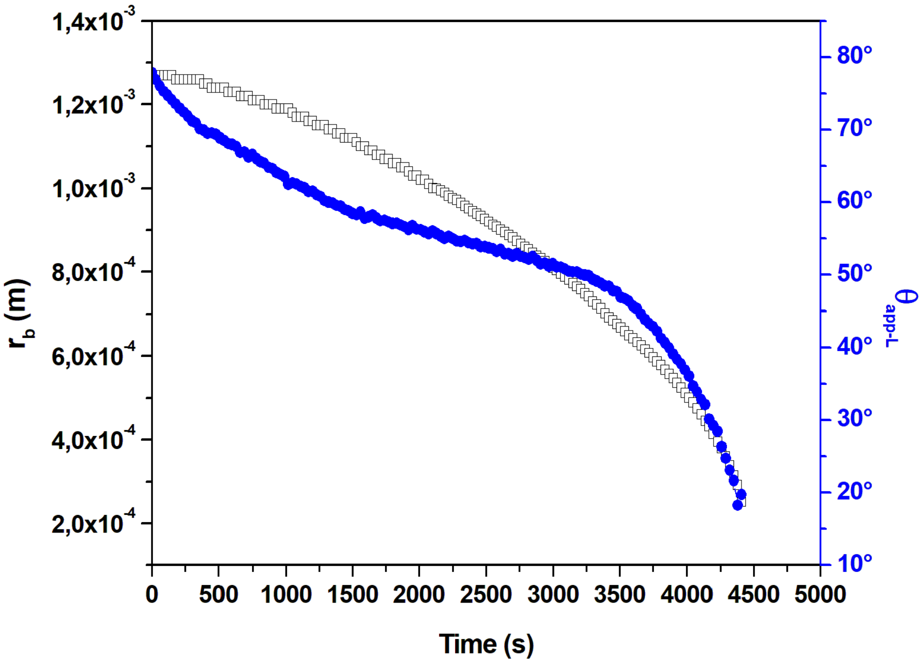

Figure S.37. The change of contact radius (r_b) and apparent contact angle at the liquid three-phase contact line at the lubricant-water-air phases ($\theta_{app\text{-}L}$) during the evaporation of a water drop placed on a SLIPS sample formed by infusing 20 cSt silicone oil on the S-3 sample with a top oil layer thickness $(h_{oil})_i$ = 13,27 μm and initial ridge height $(h_r)_i$ = 448 μm.

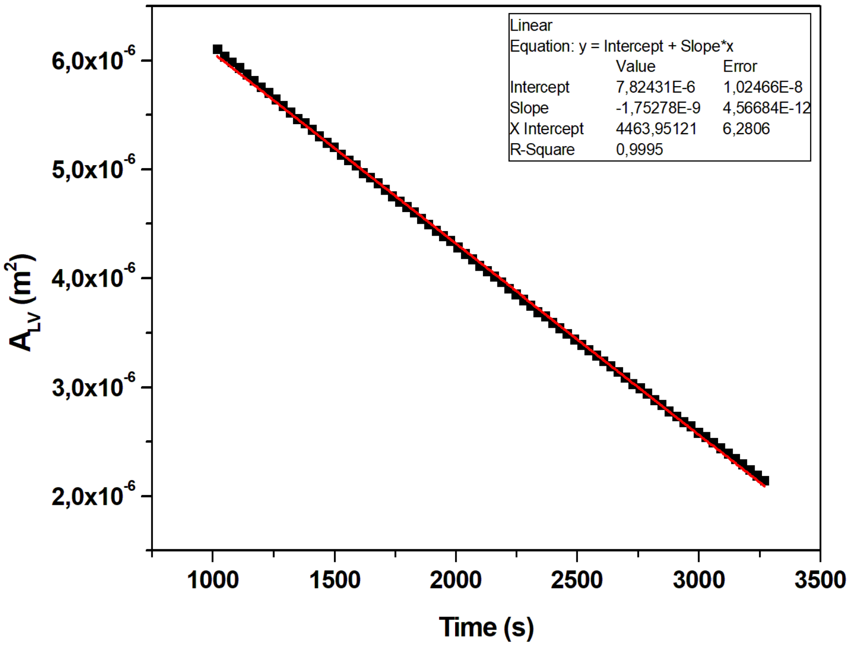

Figure S.38. The change of liquid-air interfacial area of water drop (A_{LV}) during the evaporation of a water drop on a SLIPS sample formed by infusing 20 cSt silicone oil on the S-3 sample with a top oil layer thickness $(h_{oil})_i$ = 13,27 μm and initial ridge height $(h_r)_i$ = 448 μm.

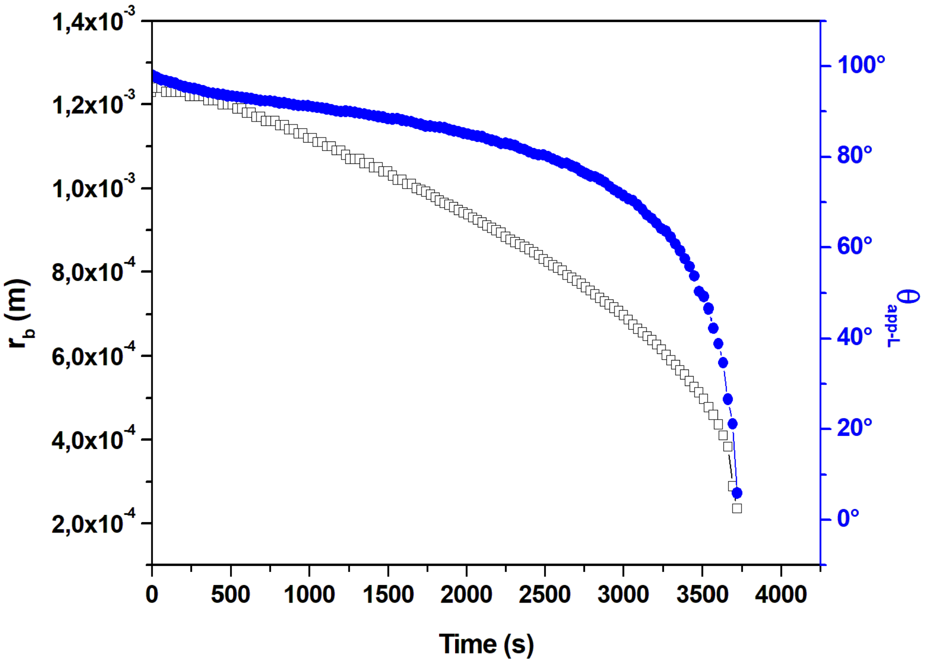

Figure S.39. The change of contact radius (r_b) and apparent contact angle at the liquid three-phase contact line at the lubricant-water-air phases ($\theta_{app\text{-}L}$) during the evaporation of a water drop placed on a SLIPS sample formed by infusing 350 cSt silicone oil on the S-3 sample with a top oil layer thickness $(h_{oil})_i$ = 0,16 μm and initial ridge height $(h_r)_i$ = 124 μm.

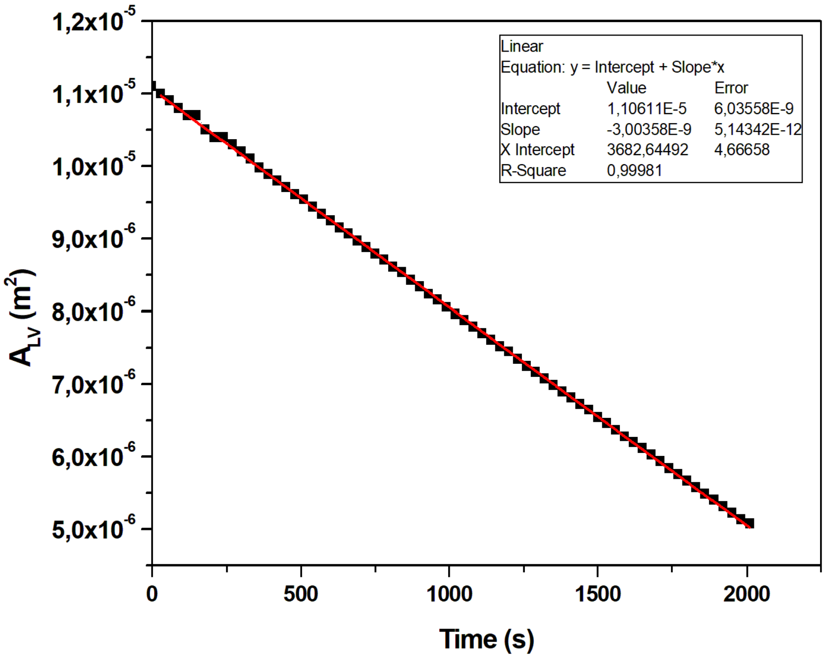

Figure S.40. The change of liquid-air interfacial area of water drop (A_{LV}) during the evaporation of a water drop on a SLIPS sample formed by infusing 350 cSt silicone oil on the S-3 sample with a top oil layer thickness $(h_{oil})_i$ = 0,16 μm and initial ridge height $(h_r)_i$ = 124 μm.

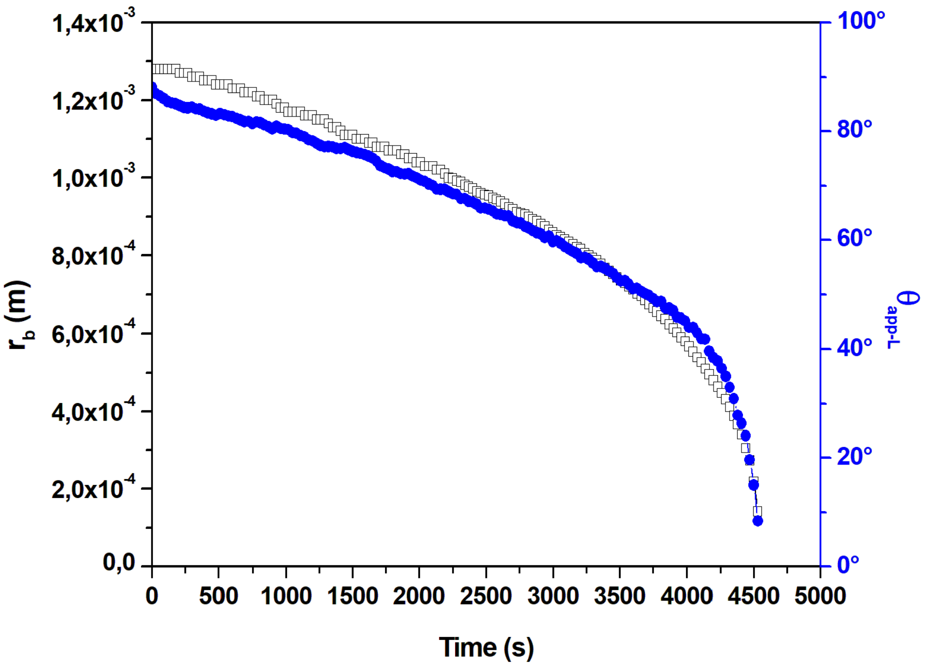

Figure S.41. The change of contact radius (r_b) and apparent contact angle at the liquid three-phase contact line at the lubricant-water-air phases (θ_{app-L}) during the evaporation of a water drop placed on a SLIPS sample formed by infusing 350 cSt silicone oil on the S-3 sample with a top oil layer thickness $(h_{oil})_i$ =5,31 μm and initial ridge height $(h_r)_i$ = 285 μm.

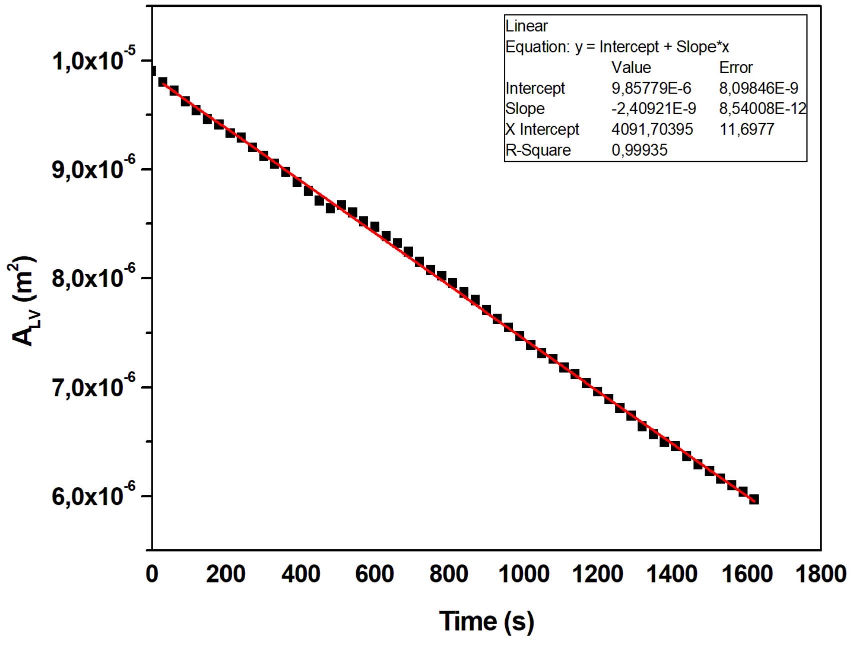

Figure S.42. The change of liquid-air interfacial area of water drop (A_{LV}) during the evaporation of a water drop on a SLIPS sample formed by infusing 350 cSt silicone oil on the S-3 sample with a top oil layer thickness $(h_{oil})_i$ = 5,31 μm and initial ridge height $(h_r)_i$ = 285 μm.

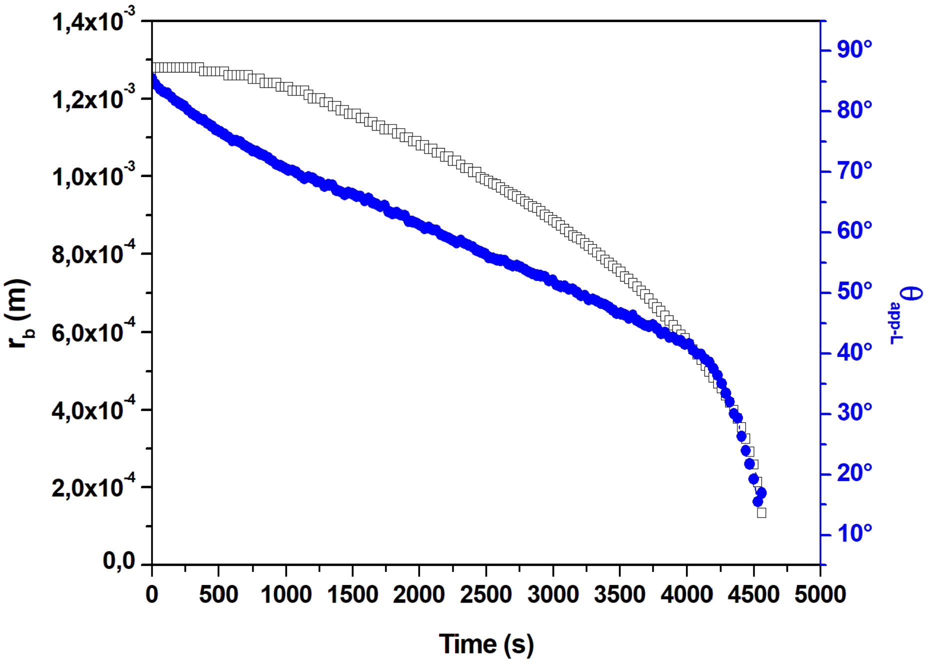

Figure S.43. The change of contact radius (r_b) and apparent contact angle at the liquid three-phase contact line at the lubricant-water-air phases ($\theta_{app\text{-}L}$) during the evaporation of a water drop placed on a SLIPS sample formed by infusing 350 cSt silicone oil on the S-3 sample with a top oil layer thickness $(h_{oil})_i$ = 7,92 μm and initial ridge height $(h_r)_i$ = 344 μm.

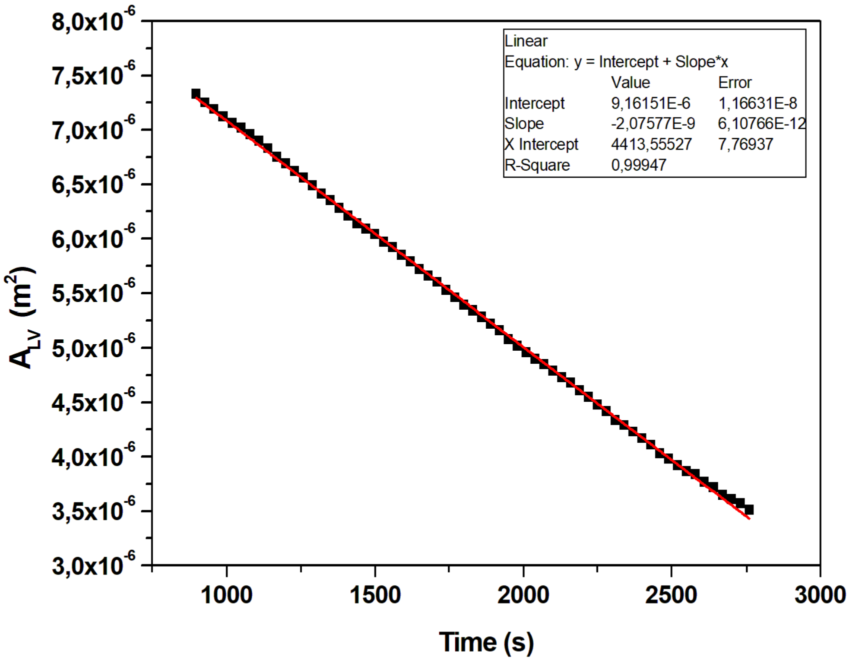

Figure S.44. The change of liquid-air interfacial area of water drop (A_{LV}) during the evaporation of a water drop on a SLIPS sample formed by infusing 350 cSt silicone oil on the S-3 sample with a top oil layer thickness $(h_{oil})_i$ = 7,92 μm and initial ridge height $(h_r)_i$ = 344 μm.

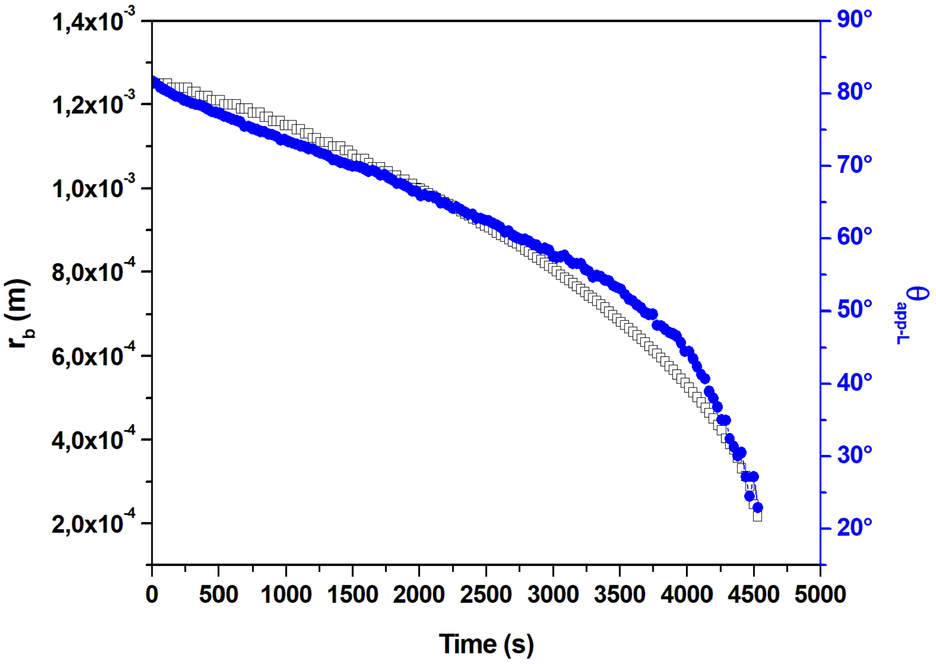

Figure S.45. The change of contact radius (r_b) and apparent contact angle at the liquid three-phase contact line at the lubricant-water-air phases ($\theta_{app\text{-}L}$) during the evaporation of a water drop placed on a SLIPS sample formed by infusing 350 cSt silicone oil on the S-3 sample with a top oil layer thickness $(h_{oil})_i$ = 11,40 μm and initial ridge height $(h_r)_i$ = 398 μm.

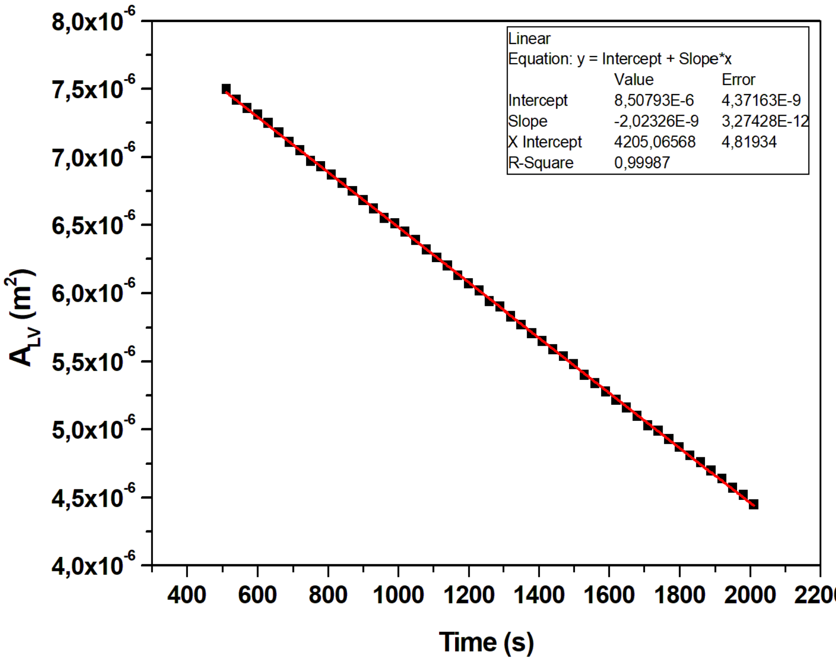

Figure S.46. The change of liquid-air interfacial area of water drop (A_{LV}) during the evaporation of a water drop on a SLIPS sample formed by infusing 350 cSt silicone oil on the S-3 sample with a top oil layer thickness $(h_{oil})_i$ = 11,40 μm and initial ridge height $(h_r)_i$ = 398 μm.
